# Supplementary figures and images for: Alternaria alternata effector AaAlta1 targets CmWD40 and participates in regulating disease resistance in Chrysanthemum morifolium
Source: PLoS Pathog. 2025 Mar 31;21(3):e1012942. doi: 10.1371/journal.ppat.1012942 (PMC11957361; doi:10.1371/journal.ppat.1012942)

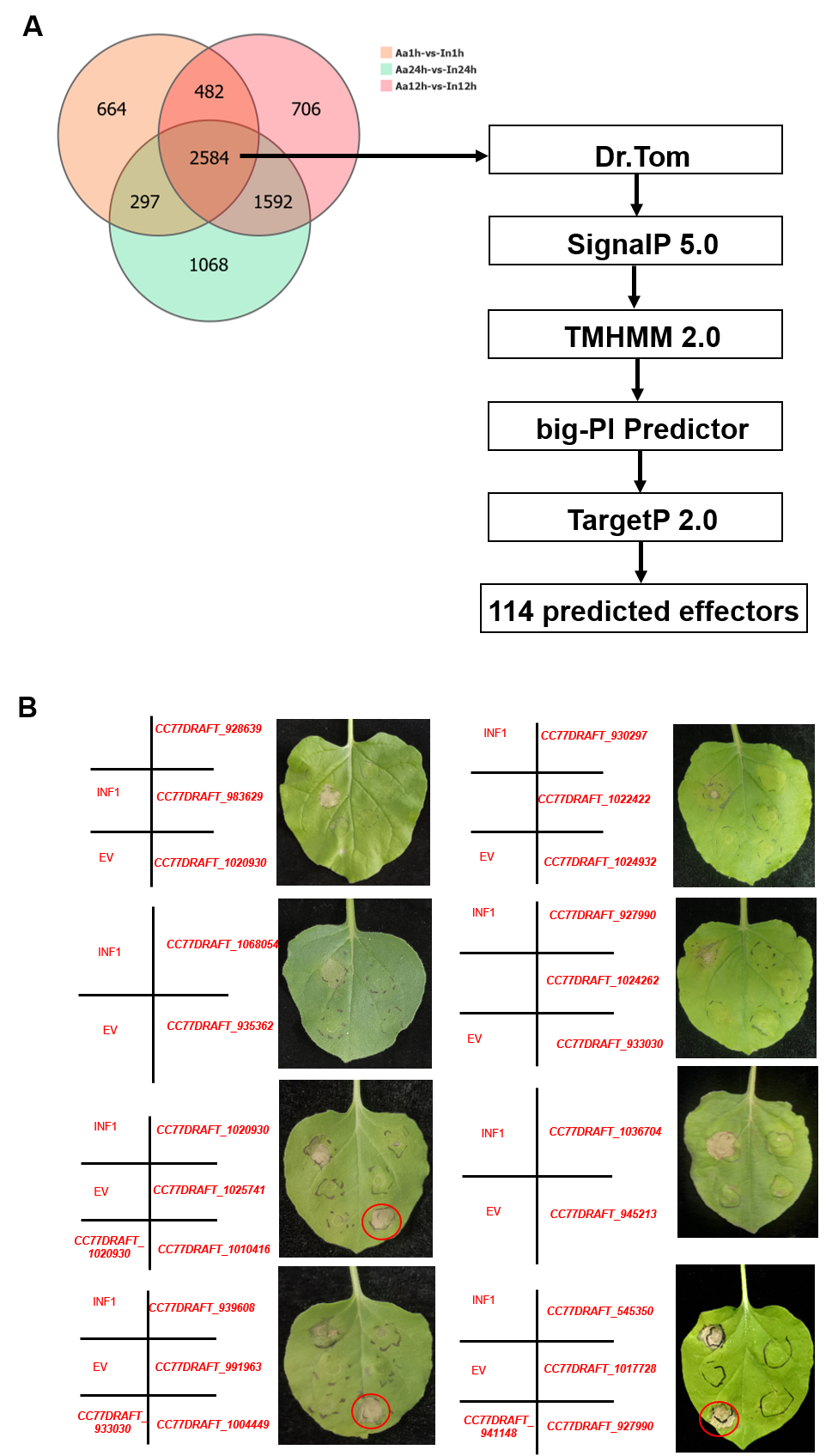

Supplement: S1 Fig — (A) High-throughput screening of candidate effectors using transcriptomics and bioinformatics analyses. (B) Transient transformation of candidate effectors in Nicotiana benthamiana leaves. Nicotiana benthamiana leaves were infiltrated with Agrobacterium tumefaciens strain GV3101 cells harboring the A. alternata effector genes, the Phytophthora infestans gene INF1 and the empty vector (EV). The Agrobacterium cells harboring the INF1 gene and EV were used as positive and negative controls, respectively. Partial results have been presented. (TIF) [file ppat.1012942.s001.tif]

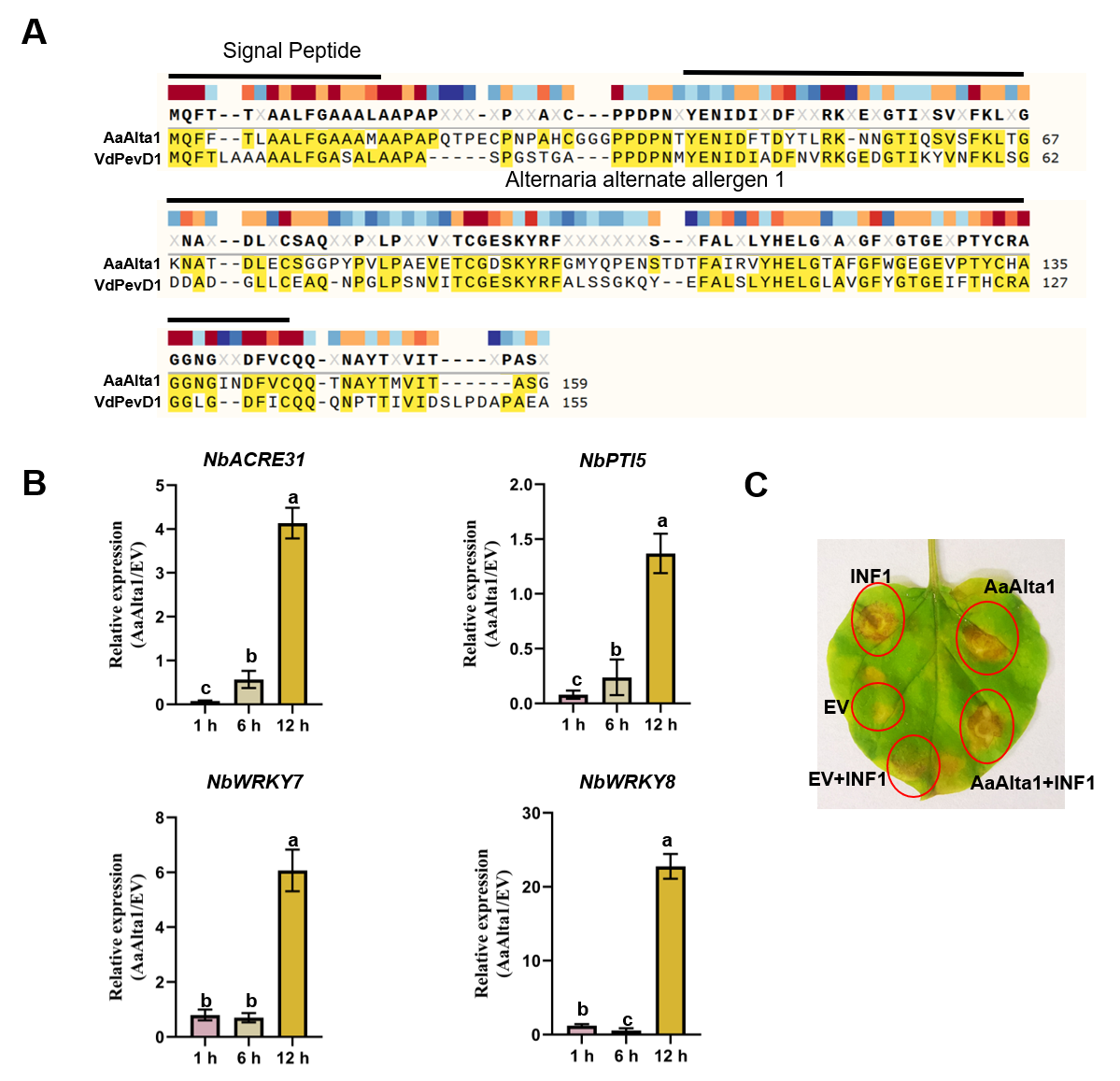

Supplement: S2 Fig — (A) Sequence alignment of the protein sequence of the AaAlta1 protein with that of its ortholog effector from Verticillium dahliae (PevD1, VDAG_02735). (B) Relative expression of immune marker genes triggered by AaAlta1 in Nicotiana benthamiana leaves. Expression levels were assessed by quantitative reverse transcriptase polymerase chain reaction. Data are presented as mean ± standard error of three biological replicates. Different letters at the top of error bars indicate significant differences (P <0.05, Tukey’s test). (C) AaAlta1 induces reactive oxygen species burst in N. benthamiana. DAB staining and decolorization were performed 3 days after the Agrobacterium treatment. The Agrobacterium strain harboring the INF1 gene was used as a positive control, and EV was used as a negative control in N. benthamiana. (TIF) [file ppat.1012942.s002.tif]

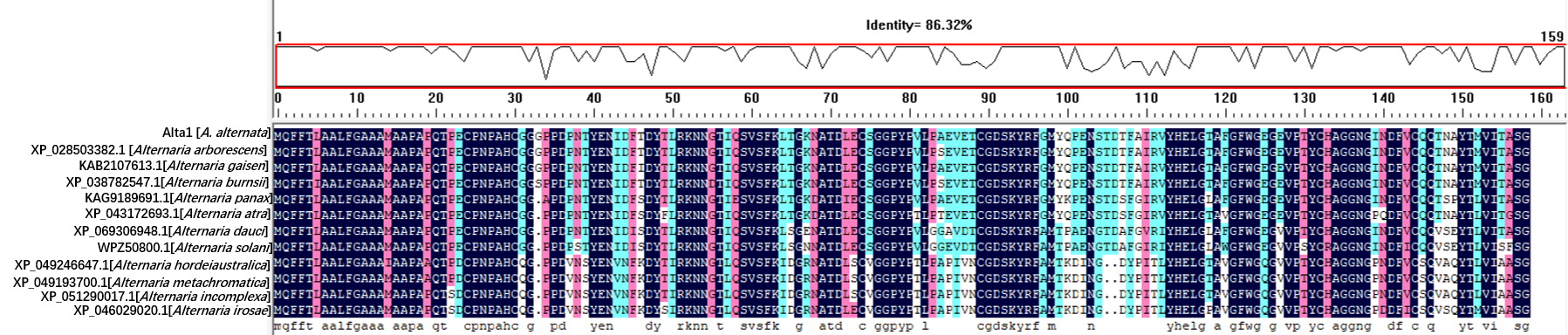

Supplement: S3 Fig — Alternaria arborescens (XP_028503382.1), Alternaria gaisen (KAB2107613.1), Alternaria burnsii (XP_038782547.1), Alternaria panax (KAG9189691.1), Alternaria atra (XP_043172693.1), Alternaria dauci (XP_069306948.1), Alternaria solani (WPZ50800.1), Alternaria hordeiaustralica (XP_049246647.10), Alternaria metachromatica (XP_049193700.1), Alternaria incomplexa (XP_051290017.1), Alternaria irosae (XP_046029020.1). (TIF) [file ppat.1012942.s003.tif]

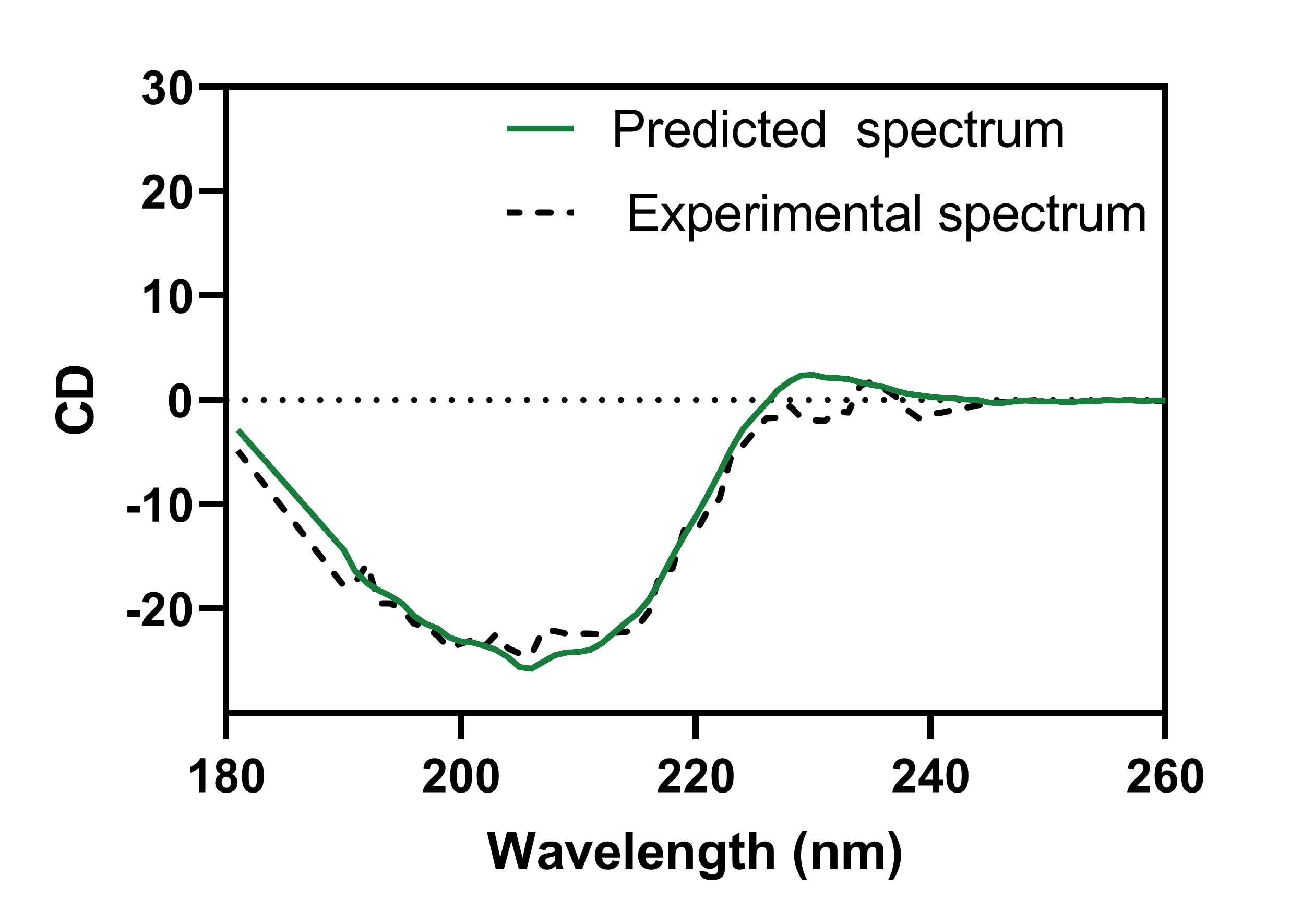

Supplement: S4 Fig — (TIF) [file ppat.1012942.s004.tif]

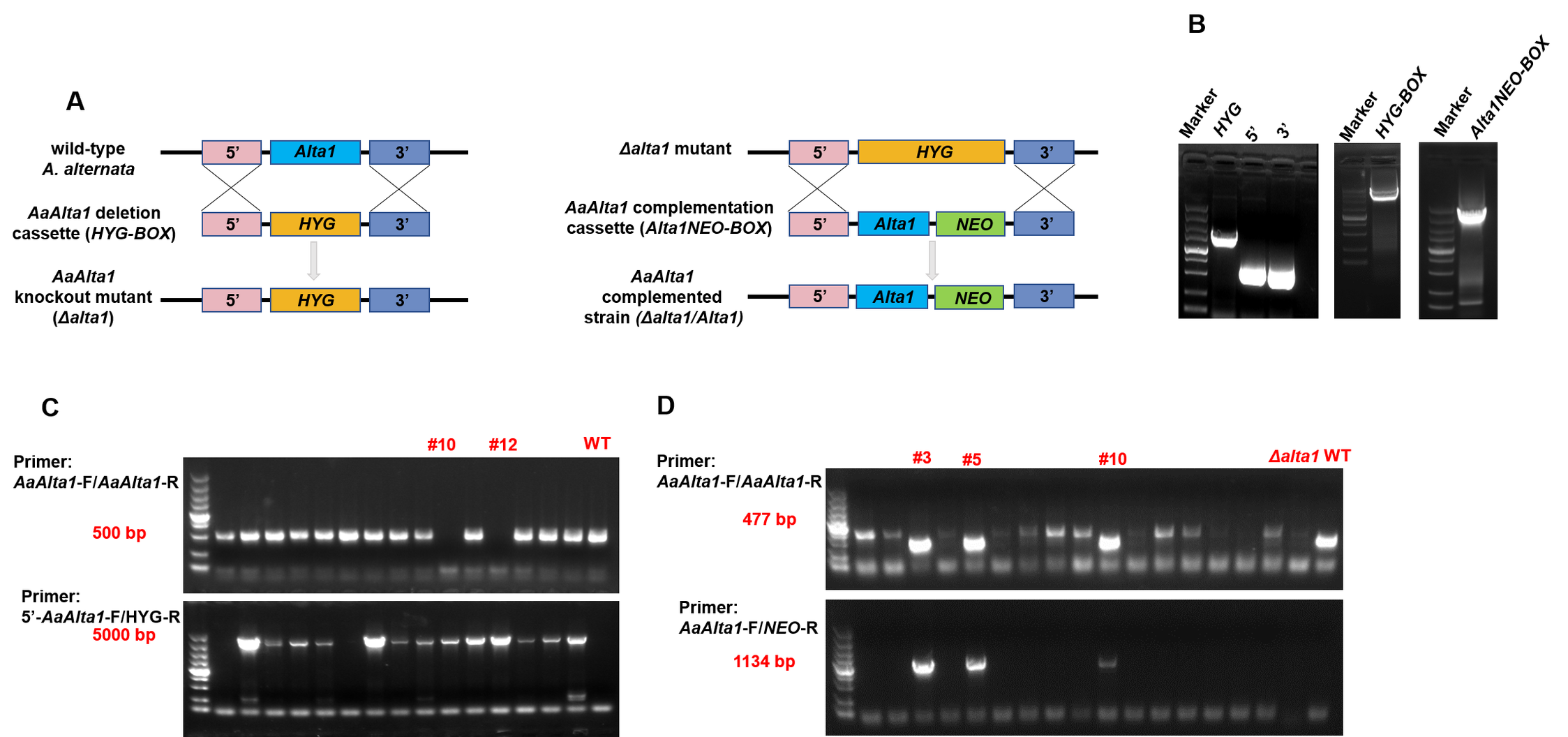

Supplement: S5 Fig — (A) Schematic diagram of the homologous recombination-based AaAlta1 KO (Δalta1) and complementation (Δalta1/Alta1) strategy. In the KO construct, the hygromycin phosphotransferase gene is flanked by 966 bp of the 5′ sequence and 812 bp of the 3′ sequence of the gene region of AaAlta1. In the complementation construct, AaAlta1 and the neomycin resistance gene were flanked by the 5′ sequence and 3′ sequence of the AaAlta1 gene region. (B) Polymerase chain reaction-based verification of the construction of replacement cassette. (C, D) WT, Δalta1, and Δalta1/Alta1 strains were identified at the DNA level. (TIF) [file ppat.1012942.s005.tif]

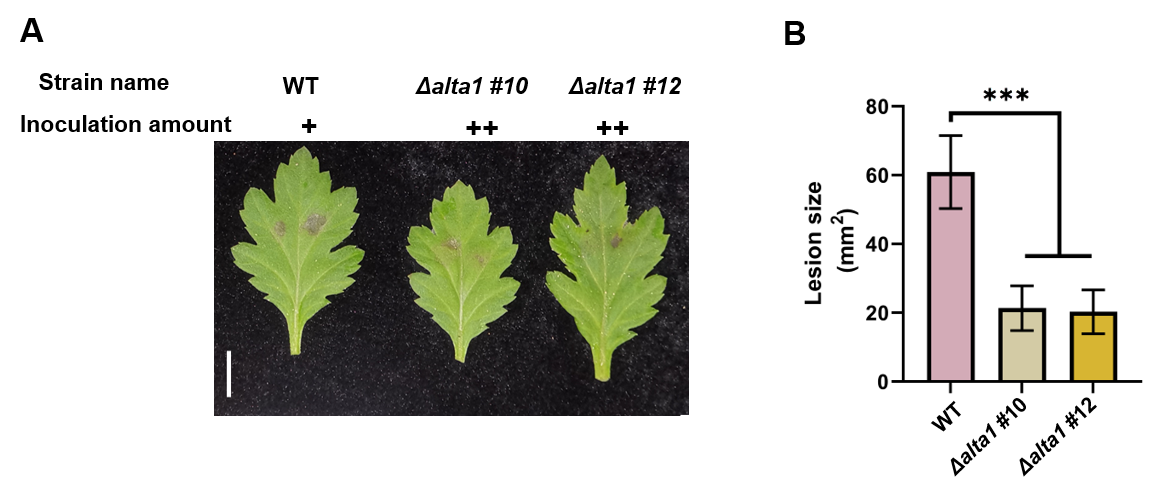

Supplement: S6 Fig — (A) Symptoms of the disease on chrysanthemum leaves inoculated with WT at mycelial normal amounts, and two Δalta1 (AaAlta1 knockout mutant) strains at mycelial double amounts. Images were captured at 48 hpi. + represents inoculated with 1 mL collected homogeneous mycelium suspension. Scale bar = 1 cm. (B) Disease severity was determined by measuring the lesion area (mm2) of leaves 48 hpi. Data are presented as the mean ± standard error of three biological replicates. *** P ≤0.0001 compared with control, as calculated by one-way analysis of variance. (TIF) [file ppat.1012942.s006.tif]

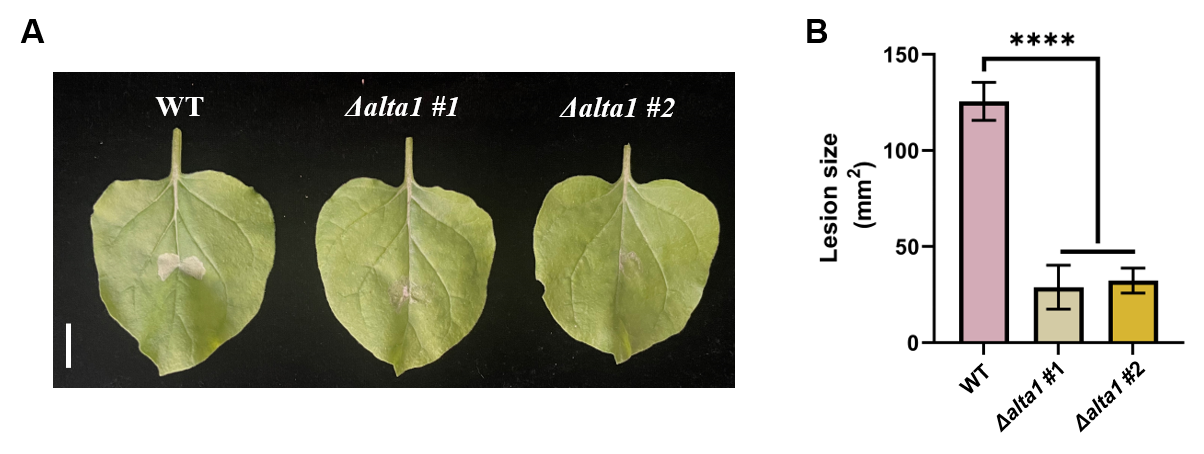

Supplement: S7 Fig — (A) Symptoms of the tobacco brown spot disease on Nicotiana benthamiana leaves inoculated with WT and Δalta1 (AaAlta1 knockout mutant) strains. Images were captured at 24 hpi. Scale bar = 1 cm. (B) Disease severity was determined by measuring the lesion area (mm2) of leaves 24 hpi. Data are presented as the mean ± standard error of three biological replicates. **** P ≤0.00001 compared with control, as calculated by one-way analysis of variance. (TIF) [file ppat.1012942.s007.tif]

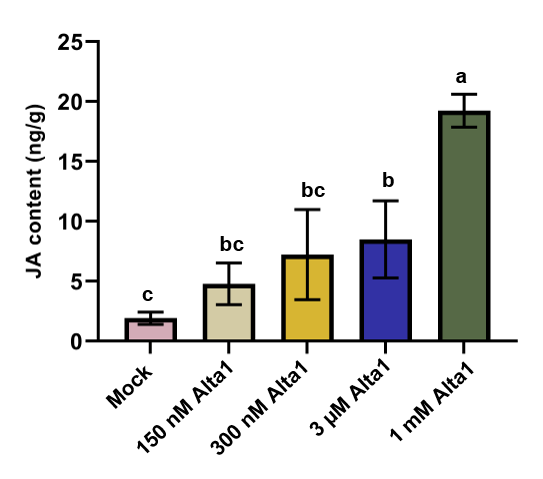

Supplement: S8 Fig — Representative chrysanthemum leaves infiltrated with purified Alta1 protein (150 nM to 1 mM) or mock solution. Measurements of JA content after 24 h. Data are presented as the mean ± standard error of three biological replicates. Different letters at the top of error bars indicate significant differences (P <0.05, Tukey’s test). (TIF) [file ppat.1012942.s008.tif]

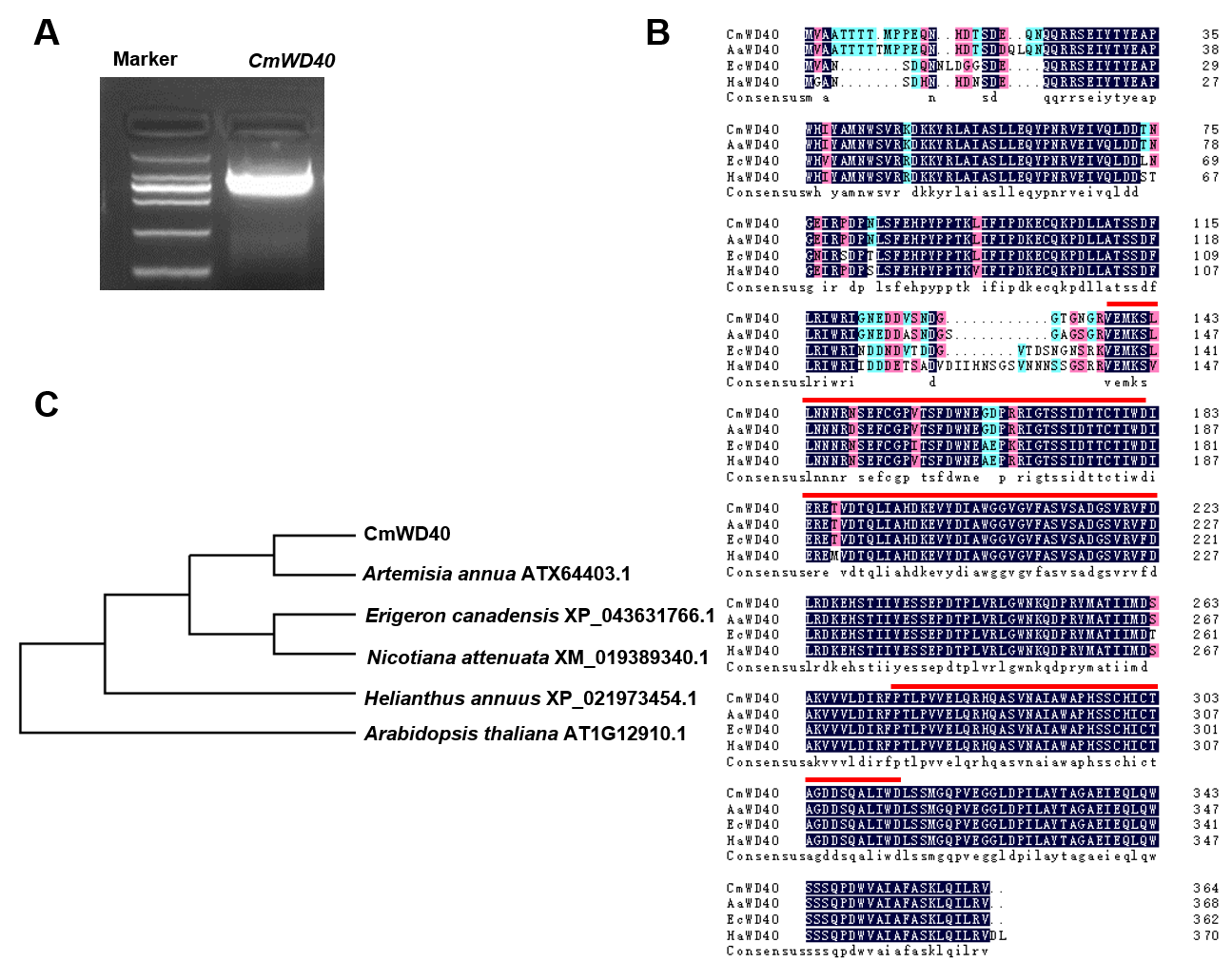

Supplement: S9 Fig — (A) Polymerase chain reaction product of the cloned CmWD40 gene. (B) Multiple sequence alignment of the CmWD40 and homologous WD40 proteins of other species, i.e., Artemisia annua (AaWD40, ATX64403.1), Erigeron canadensis (EcWD40, XP_043631766.1), Helianthus annuus (HaWD40, XP_021973454.1). The WD40 repeat domain is marked with red lines. (C) Phylogenetic tree of the CmWD40 and other WD40 proteins, including Artemisia annua, Helianthus annuus, Erigeron canadensis, Nicotiana attenuata (NaLWD1, XM_019389340.1), and Arabidopsis thaliana (AtLWD1). (TIF) [file ppat.1012942.s009.tif]

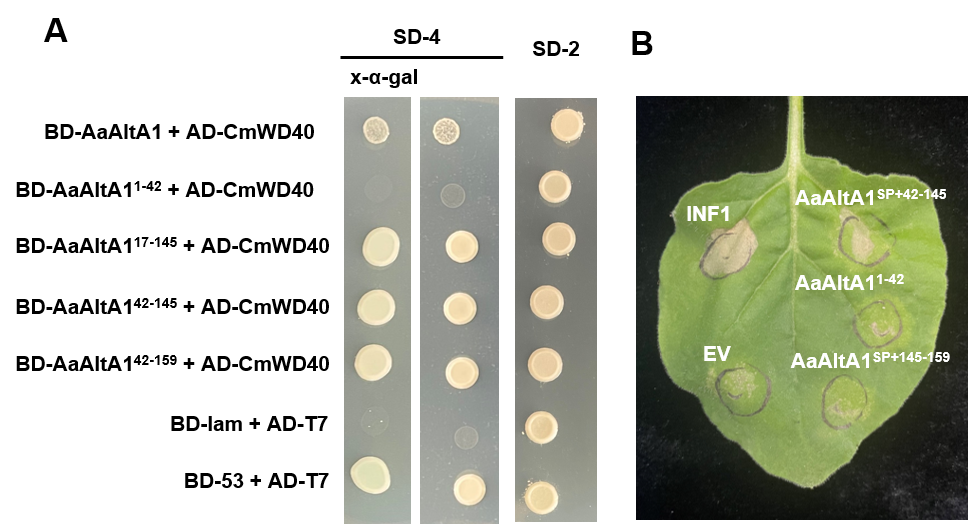

Supplement: S10 Fig — SD-4, SD/-His/-Leu/-Trp/-Ade- medium; SD-2, SD/-Leu/-Trp- medium. (B) Images showing the phenotypes of leaves infiltrated with Agrobacterium strains harboring constructs encoding the different deletion fragments of AaAlta1 3 days after infiltration. (TIF) [file ppat.1012942.s010.tif]

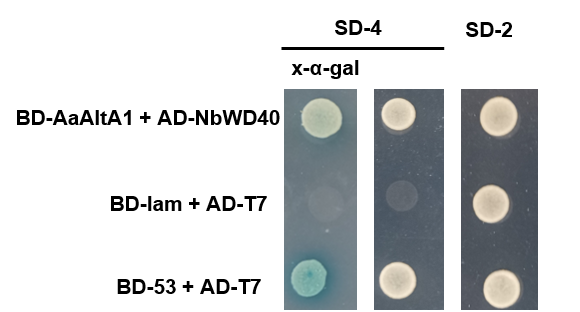

Supplement: S11 Fig — SD-4, SD/-His/-Leu/-Trp/-Ade- medium; SD-2, SD/-Leu/-Trp- medium. (TIF) [file ppat.1012942.s011.tif]

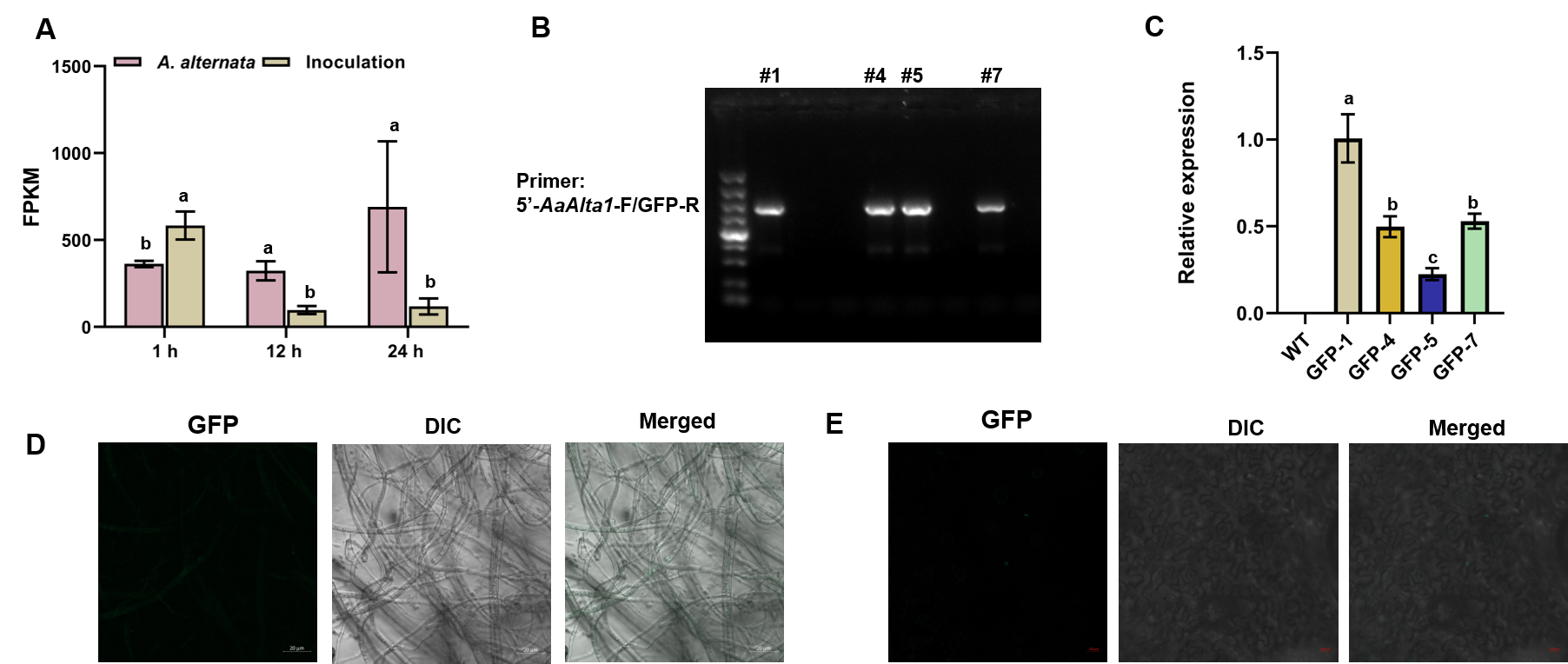

Supplement: S12 Fig — (A) Identification of Alta1-GFP fusion strains at the DNA level. (B) Relative expression of GFP in different fluorescent strains. (C) Confocal microscopy images showing fluorescent hypha in the Alta1-GFP fusion strain. (D) Subcellular localization of the fusion protein in the Alta1-GFP fusion strain. (TIF) [file ppat.1012942.s012.tif]

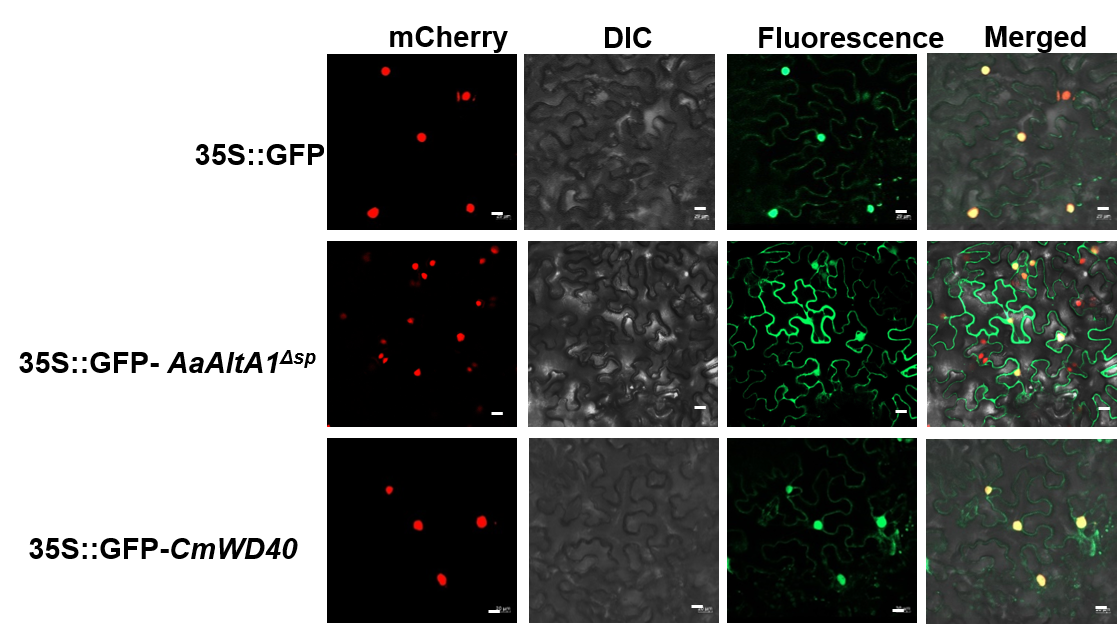

Supplement: S13 Fig — The co-expressed 35S::D53-RFP (red fluorescence protein) construct was used as a nuclear marker. Marker: images taken in the red fluorescence channel; GFP: green fluorescent protein (image captured in the green fluorescence channel); DIC: differential interference contrast (image captured in the bright light channel); merged: both overlay plots. Scale bars = 20 μm. (TIF) [file ppat.1012942.s013.tif]

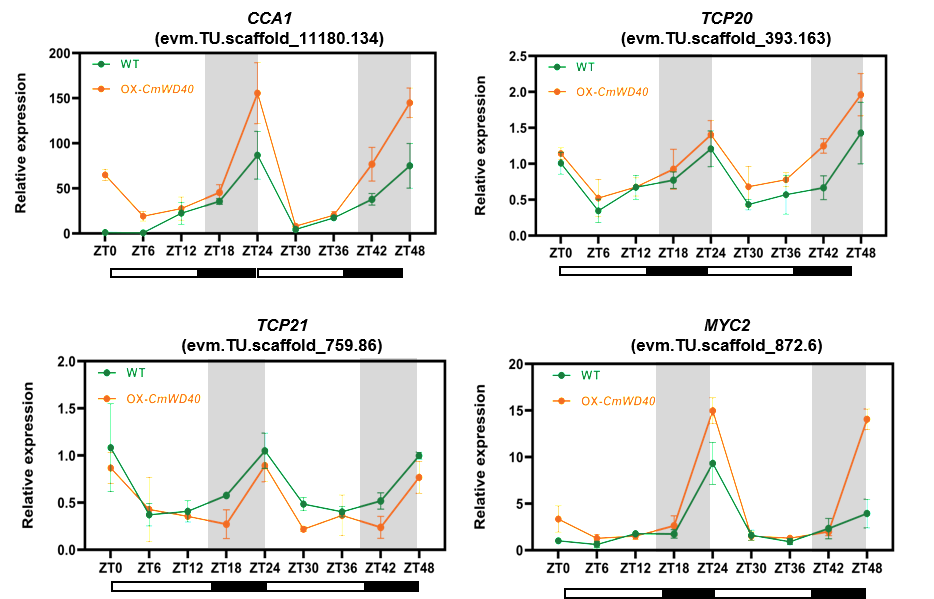

Supplement: S14 Fig — Chrysanthemum plants were cultured in LD conditions for 4 weeks, and samples were collected every 6 h at the indicated times. Gene expression levels were assessed by quantitative reverse transcriptase polymerase chain reaction. Data are presented as mean ± standard error of three biological replicates. (TIF) [file ppat.1012942.s014.tif]

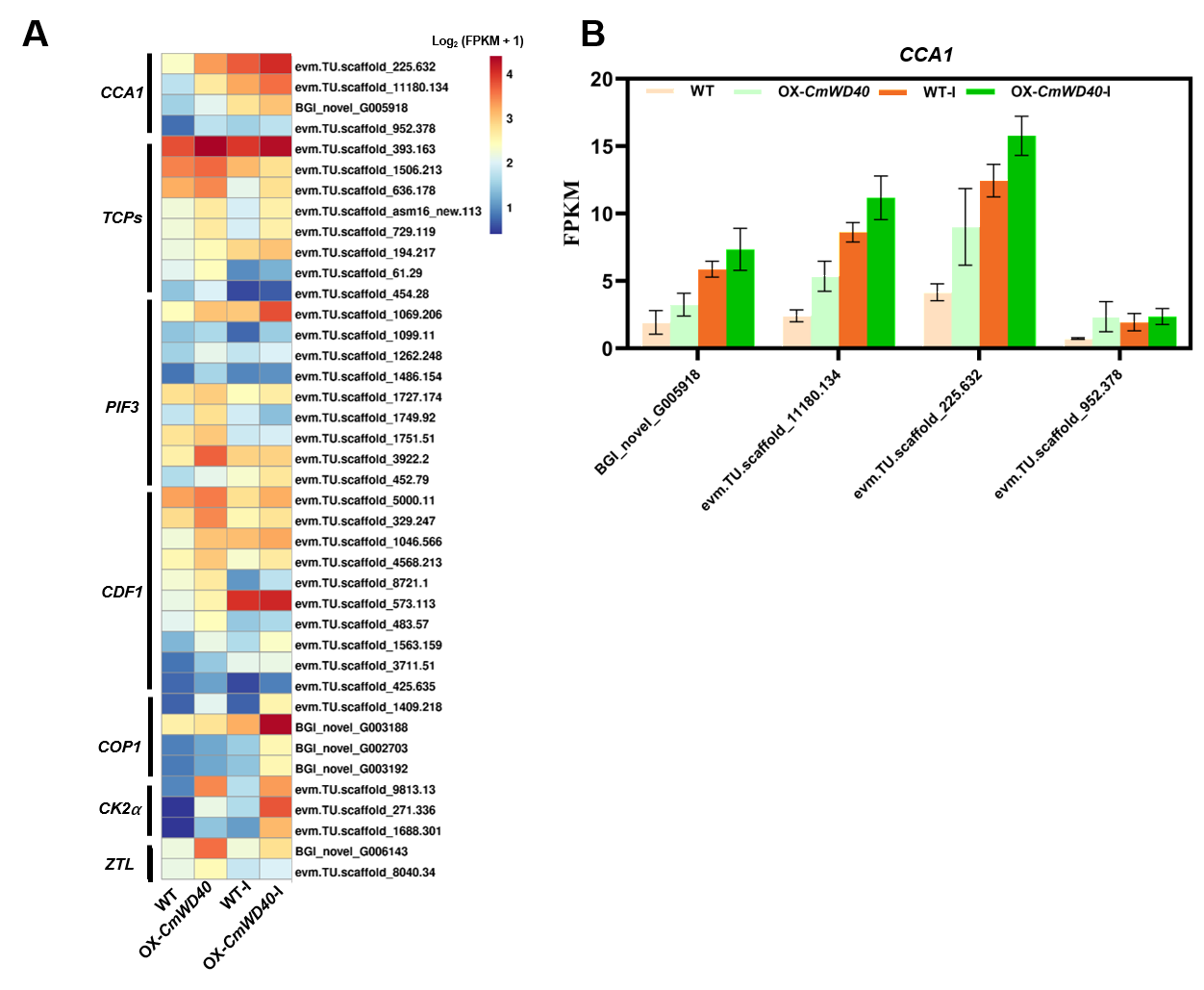

Supplement: S15 Fig — (A) Heatmaps of differentially expressed genes (DEGs) encoding clock-related genes in overexpression (OX)-CmWD40 transgenic chrysanthemum, as assessed using RNA-seq. The gene expression values are normalized log2(FPKM [fragments per kilobase of transcript per million fragments mapped] + 1). From left to right are shown WT, OX-CmWD40, WT-I, and OX-CmWD40-I. WT, control, non-infected plants (WT cultivar ‘Jinba’); OX-WD40, non-infected OX-CmWD40 transgenic plants; WT-I, control, infected plants; OX-WD40-I, infected OX-CmWD40 transgenic plants. (B) FPKM of CCA1 in RNA-Seq. (TIF) [file ppat.1012942.s015.tif]

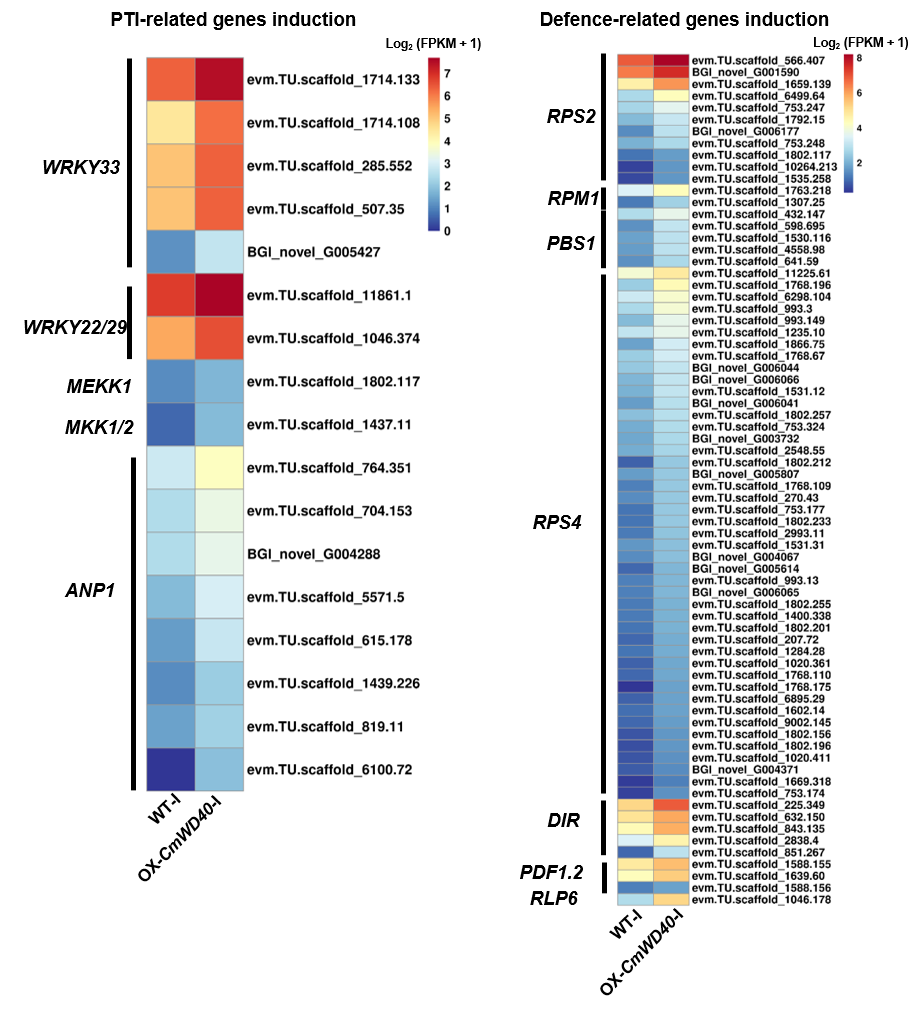

Supplement: S16 Fig — The gene expression values are normalized log2(FPKM [fragments per kilobase of transcript per million fragments mapped] + 1). From left to right are shown WT-I, OX-CmWD40-I. WT-I, control, infected plants; OX-WD40-I, infected OX-CmWD40 transgenic plants. (TIF) [file ppat.1012942.s016.tif]

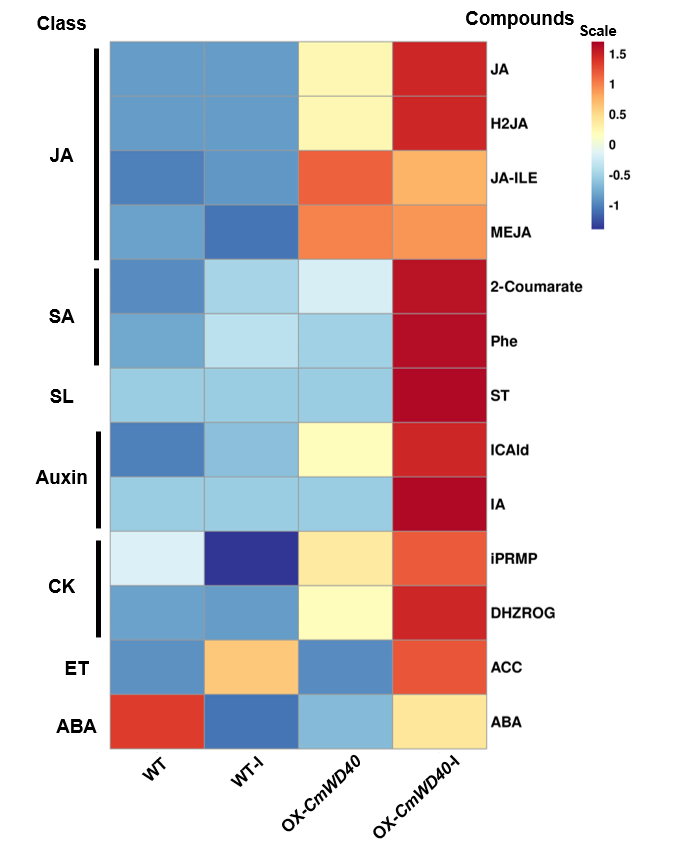

Supplement: S17 Fig — The horizontal axis represents the names of samples, whereas the vertical axis represents the information of metabolites. Different colors indicate different content levels, with the colors filled based on the Z-score normalized values (red represents high content, and green represents low content). Cluster analysis was performed on the metabolites, with the clustering lines on the left side of the figure representing the metabolite clustering lines. (TIF) [file ppat.1012942.s017.tif]

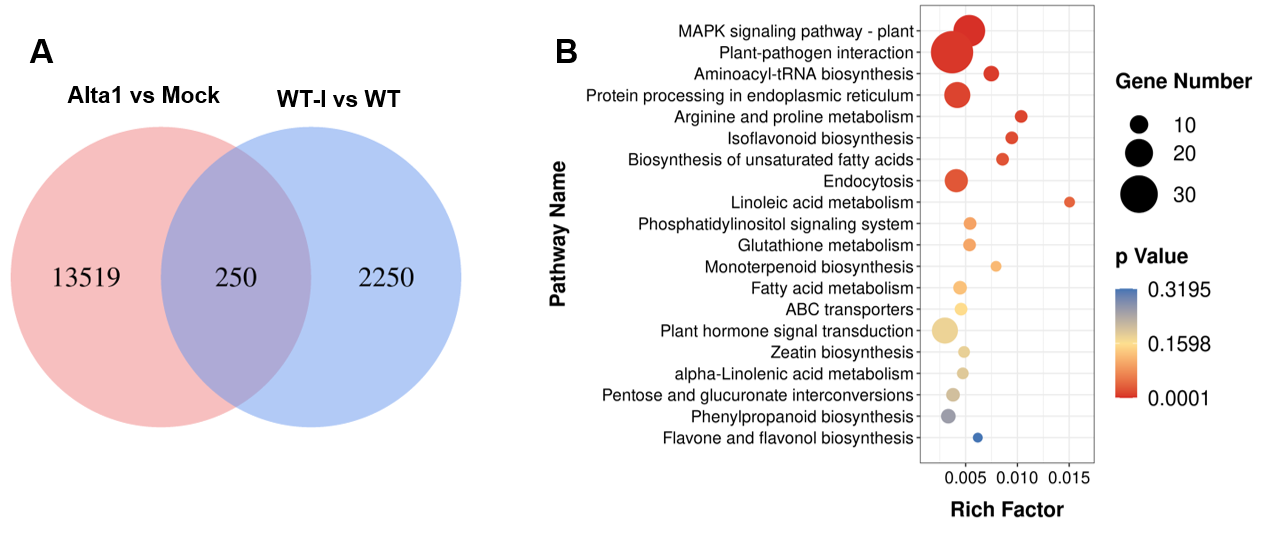

Supplement: S18 Fig — (A) Venn diagram analysis of the DEGs induced by Alta1 and A. alternata. (B) Kyoto Encyclopedia of Genes and Genomes enrichment analysis of the DEGs co-upregulated by Alta1 and A. alternata. (TIF) [file ppat.1012942.s018.tif]

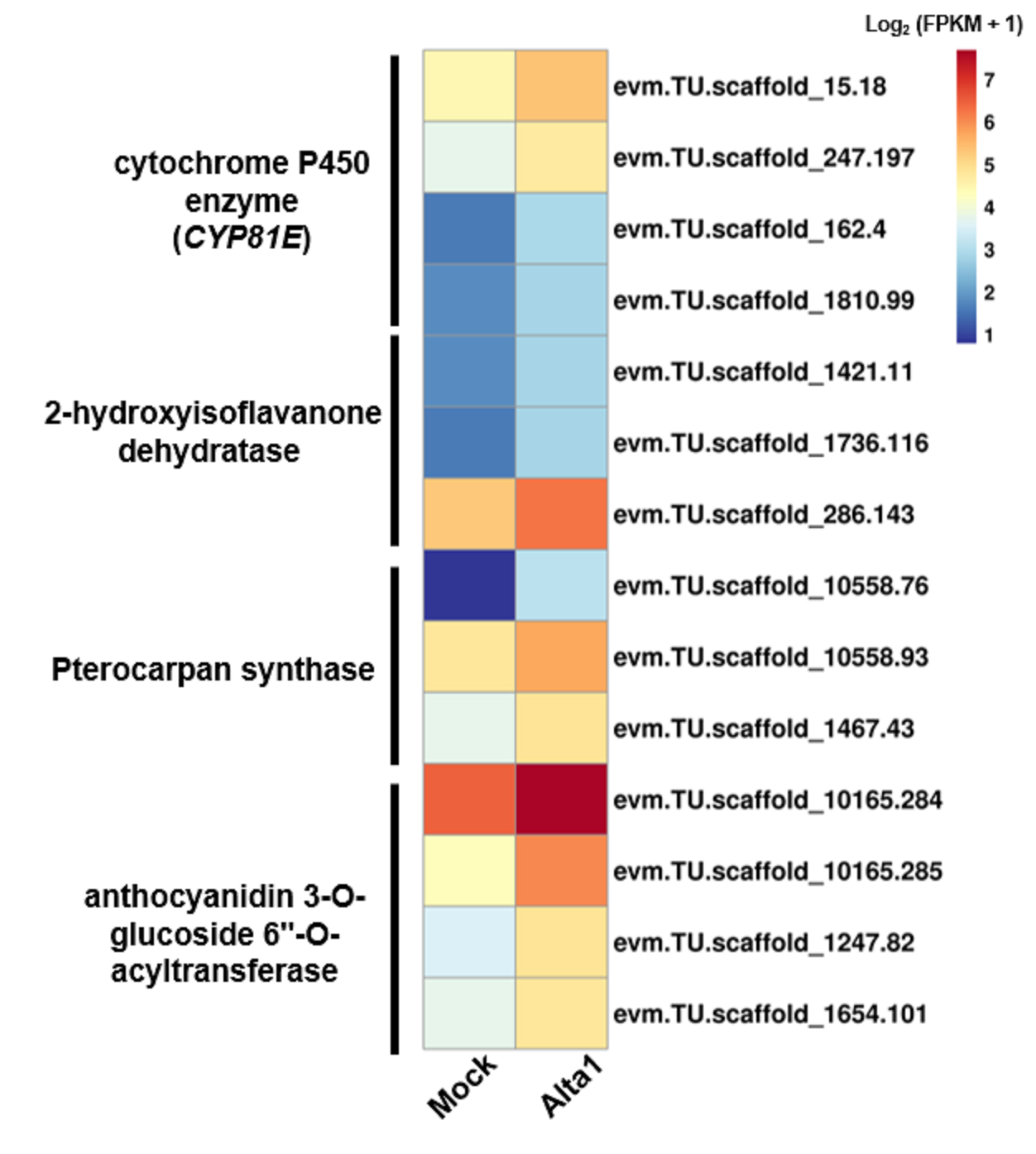

Supplement: S19 Fig — The gene expression values are normalized log2 (FPKM [fragments per kilobase of transcript per million fragments mapped] + 1). From left to right are shown the mock and Alta1. Mock, heat map of the DEGs in mock-infiltrated tissues; Alta1, heat map of the DEGs in 300 nM Alta1-infiltrated tissues. (TIF) [file ppat.1012942.s019.tif]
