# Supplementary material for: Alternaria alternata effector AaAlta1 targets CmWD40 and participates in regulating disease resistance in Chrysanthemum morifolium
Source: PLoS Pathog. 2025 Mar 31;21(3):e1012942. doi: 10.1371/journal.ppat.1012942 (PMC11957361; doi:10.1371/journal.ppat.1012942)
Supplement: Attachment — Submitted filename: Response to reviewers.docx [file ppat.1012942.s022.docx]

Dear. Dr. Thomma,

Thank you very much for sending our manuscript for peer review. At the same time, we are very grateful to the reviewer for carefully reviewing our manuscript and putting forward the valuable comments and suggestions.

After carefully reading the reviewer's comments, we find that the comments are of great help for the improvement of our manuscript. In the past days, we have made great efforts to carefully revise the manuscript according to these comments. All of the comments have been considered in the revised manuscript, and all of the revisions are marked in red in the revised version.

Below we have provided in detail the point-by-point replies to the reviewer’s comments. Now we are resubmitting the revised manuscript to ***PLOS Pathogens***, and sincerely hope that it can be finally recommended for publication in this journal after the further evaluation.

Sincerely,

Corresponding author:

Jiafu Jiang

Email: jiangjiafu@njau.edu.cn

-----------------------------------------------------------------------------------------------------

REVIEWER COMMENTS:

**Part II – Major Issues**

Reviewer #1:

1. Do a genome scan for this locus in other A. alternata strains - see how prevalent it is within the species and do MSA to show homology. How well conserved is this gene and encoded protein?

A: Thank you for your suggestion. We have done the comparison of homologous proteins in other *A. alternata* strains by MSA (S3 Fig). We found the homology of these proteins is higher than 86%, suggesting that this gene and its encoded protein is high conserved (lines 131-136 in the new revision).

S3 Fig. Multiple sequence alignment of *Aa*Alta1 with 11 orthologs from other *Alternaria alternata* strains. *Alternaria arborescens* (XP_028503382.1), *Alternaria gaisen* (KAB2107613.1), *Alternaria burnsii* (XP_038782547.1), Alternaria panax (KAG9189691.1), *Alternaria atra* (XP_043172693.1), *Alternaria dauci* (XP_069306948.1), Alternaria solani (WPZ50800.1), *Alternaria hordeiaustralica* (XP_049246647.10), *Alternaria metachromatica* (XP_049193700.1), *Alternaria incomplexa* (XP_051290017.1), *Alternaria irosae* (XP_046029020.1).

2. Do functional work in at least 1 additional strain of the fungus - perhaps a type specimen or NRRL strain.

A: Thank you for your suggestion. We selected an additional strain of *A. alternata* that causes tobacco brown spot for functional work (S6 Fig). Two *Δalta1* mutant strains (*Δalta1 #1* and *#2*) both produced mild disease symptoms on *N. benthamiana* leaves compared to the wild-type strain, with the lesion area being approximately reduced by 77% and 74%, respectively. These results indicate that the function of Alta1 is conserved (lines 230-236 in the new revision).

S6 Fig. Alta1 is important for the full virulence expression of *Alternaria alternata* (tobacco pathotype). (A) Symptoms of the tobacco brown spot disease on *Nicotiana benthamiana* leaves inoculated with WT and *Δalta1* (*Aa*Alta1 knockout mutant) strains. Images were captured at 24 hpi. Scale bar = 1 cm. (B) Disease severity was determined by measuring the lesion area (mm2) of leaves 24 hpi. Data are presented as the mean ± standard error of three biological replicates. **** *P* ≤ 0.00001 compared to control, as calculated by one-way ANOVA.

3. Generate additional mutant strains for current and additional strain, and include complemented, ectopic and non-complemented controls strains in analysis.

A: Thank you for your suggestion. We have generated a second effector deletion mutant and tested it (Figs 2 and S5 Fig). The colony morphology of the mutant, complemented, and non-complemented control strains were analyzed, the virulence in the WT strains was confirmed. Additionally, we have made corresponding modifications to the legends and the manuscript (lines 215-217 in the new revision).

Fig 2. *Aa*Alta1 is important for full virulence for *A. alternata*.

S5 Fig. Generation and identification of *Aa*Alta1 knockout (KO) mutant and complementation strains.

4. Consider altering the model diagram to include other aspects of fungal infection and plant defense as it appears way too simplistic based on the conclusions of the current investigation.

A: Thank you for your suggestion. We have altered the model diagram (Fig 7D).

Fig 7. *Cm*WD40-mediated Alta1-induced disease resistance relies on MYC2.

5. The paper would also benefit from using a targeted metabolomics approach to verify JA and other phytohormone involvement at the chemical level.

A: Thank you for your suggestion. A targeted metabolomics analysis was performed on the leaves of OX-*Cm*WD40 lines (OX-#2) and WT plants with or without *A. alternata* inoculation (S16 Fig). Consistent with the LC-MS analysis results, the content of JA significantly increased in the OX-*Cm*WD40 lines. We further validated that JA is involved in the resistance induced by *Cm*WD40. Notably, apart from several JA derivatives, (±) Strigol (ST) also exhibit similar accumulation patterns in the OX-*Cm*WD40 lines. The accumulation of Aminocyclopropanecarboxylic acid (ACC) also shows significant differences in different treatments, suggesting that other phytohormone are also involved. ACC can be oxidized by ACC oxidases to produce ethylene (ET) [68]. The production of ET can enhance the resistance of litchi to *Peronophythora litchi* [69]. ET can also regulate plant resistance to necrotrophic pathogens, such as *Botrytis cinerea* [70]. Taken together, these data illustrate that the JA and other phytohormones can be activated by *Cm*WD40 to trigger plant immune responses (lines 474-476, 639-648, in the new revision).

[68] Park C, Lee HY, Yoon GM. The regulation of ACC synthase protein turnover: a rapid route for modulating plant development and stress responses. Curr Opin Plant Biol. 2021;63:102046. Epub 2021/05/10. doi: 10.1016/j.pbi.2021.102046. PubMed PMID: 33965697.

[69] Li P, Li W, Zhou X, Situ J, Xie L, Xi P, et al. Peronophythora litchii RXLR effector P. litchii avirulence homolog 202 destabilizes a host ethylene biosynthesis enzyme. Plant Physiol. 2023;193(1):756-74. Epub 2023/05/26. doi: 10.1093/plphys/kiad311. PubMed PMID: 37232407.

[70] Zhao Y, Wei T, Yin KQ, Chen Z, Gu H, Qu LJ, et al. Arabidopsis RAP2.2 plays an important role in plant resistance to Botrytis cinerea and ethylene responses. New Phytol. 2012;195(2):450-60. Epub 2012/04/26. doi: 10.1111/j.1469-8137.2012.04160.x. PubMed PMID: 22530619.

S16 Fig. Heatmaps analysis of metabolites. The horizontal axis represents the names of samples, whereas the vertical axis represents the information of metabolites. Different colors indicate different content levels, with the colors filled based on the *Z*-score normalized values (red represents high content, and green represents low content). Cluster analysis was performed on the metabolites, with the clustering lines on the left side of the figure representing the metabolite clustering lines.

Reviewer #2: (No Response)

Reviewer #3: The first and major remark is that the manuscript would benefit from an extensive grammatical cleanup and from a more accurate use of references.

A: Thank you for your suggestion. The grammatical errors and typos have been corrected by an English native speaker. We have carefully checked and updated references throughout.

Secondly, a second effector deletion mutant should be tested. The authors used two complemented isolates but only one deletion mutant.

A: We have generated a second effector deletion mutant and tested it (Figs 2 and S5 Fig). The colony morphology of the mutant, complemented, and non-complemented control strains were analyzed, the virulence in the WT strains was confirmed. Additionally, we have made corresponding modifications to the figure (Figs 2 and S5 Fig), legends and the manuscript. Please see in lines 215-217.

Fig 2. *Aa*Alta1 is important for full virulence for *A. alternata*.

S5 Fig. Generation and identification of *Aa*Alta1 knockout (KO) mutant and complementation strains.

Thirdly, the question how the interaction of Alta1 effector with WD40 can trigger cell death was not addressed. Is this interaction required to trigger cell death or does it occur via a different mechanism? Could a certain JA gradient might be responsible for the observed cell death response upon effector infiltration? Was the protein conformation investigated with crystallography/CD spectroscopy to ensure that the purified protein used for infiltration assay does match the in silico predicted effector protein structure?

A: Thank you for your comment. We determined the content of JA after infiltrating chrysanthemum leaves with a gradient of different concentrations of Alta1 protein. The results indicated that with the increase in the concentration of Alta1-treated, the production of JA gradually increased (S7 Fig). This suggests that Alta1-induced cell death and immune responses are dependent on the production of JA. Please see in lines 290-293.

S7 Fig. Measurements of jasmonic acid (JA) content in chrysanthemum leaves infiltrated with Alta1 protein. Representative chrysanthemum leaves infiltrated with purified Alta1 protein (150 nM to 1 mM) or mock solution. Measurements of JA content after 24 h. Data are presented as the mean ± standard error of three biological replicates. Different letters at the top of error bars indicate significant differences (*P*< 0.05, Tukey’s test).

The KCD web server (<https://kcd.cinvestav.mx/> ) was used to predict the CD spectra of the Alta1 protein. Crystallography/CD spectroscopy of purified Alta1 protein were measured using a circular dichroism spectrometer. The results display the spectrum calculated by KCD superimposed on the experimental spectrum (S4 Fig), ensuring that the purified protein used for the infiltration assay matches the in silico predicted effector protein structure. Please see in lines 170-173.

S4 Fig. Comparison of the experimental spectrum (black dashed line) and the predicted spectra produced using the (green line) KCD servers for the Alta1 protein.

Finally, to confirm that during the real host-pathogen interaction, the effector is delivered by the fungus to the assumed (sub-)cellular compartment, an additional experiment is required. Would it be possible to perform an in vivo inoculation assay with a fungus expressing the effector fused to a reporter (e.g. fluorescence marker such as GFP) to check effector delivery and sub-cellular localization in N. benthamiana and/or Chrysanthemum?

A: Thank you for your suggestion. We successfully constructed fungus expressing an effector fused to a reporter gene GFP, qRT-PCR analysis proved that the GFP was expressed normally in fungus (S11 Fig), but we did not observe green fluorescence after in vivo inoculation experiments. We analyzed the expression changes of Alta1 in mycelium infected or not infected from our previously published transcriptome data [28], and found that the expression level of Alta1 was lower after infection. Confocal microscopy images showed that the fluorescence of the Alta1-GFP fusion strain was very weak, which also resulted in the absence of fluorescence observed in plant cells after infection. It is possible that the expression level of Alta1 is too low, which requires further experimental verification. While sub-cellular localization in *N. benthamiana* indicate that Alta1ΔSP is localized to the nucleus and cell membrane after entering plant cells (S12 Fig). Please see in lines 337-343.

S11 Fig. Identification of the Alta1-GFP (green fluorescent protein) fusion strains. (A) The relative expression level change of Alta1 by RNA-seq. *A. alternata*, only *A. alternata* mycelium; Inoculation, contain chrysanthemum leaves and *A. alternata* mycelium. (B) Identification of Alta1-GFP fusion strains at the DNA level. (C) Relative expression of *GFP* in different fluorescent strains. (D) Confocal microscopy images showing fluorescent hypha in the Alta1-GFP fusion strain. (E) Subcellular localization of the fusion protein in the Alta1-GFP fusion strain.

[28] Liu L, Chen F, Chen S, Fang W, Liu Y, Guan Z. Dual species dynamic transcripts reveal the interaction mechanisms between *Chrysanthemum morifolium* and *Alternaria alternata*. BMC Genomics. 2021;22(1):523. Epub 2021/07/11. doi: 10.1186/s12864-021-07709-9. PMID: 34243707; PubMed Central PMCID: PMCPMC8268330.

**Part III – Minor Issues: Editorial and Data Presentation Modifications**
Please use this section for editorial suggestions as well as relatively minor modifications of existing data that would enhance clarity.

Reviewer #1: I did not find anything glaring.

Reviewer #2: Minor:

- Change A. alternate to A. alternata throughout

A: Thank you for your comments. We have changed *A. alternate* to *A. alternata* throughout.

- Correct grammatical errors and typos throughout – but can be done in editing stage.

Introduction:

-Please refocus the introduction to talk about the discovery of the allergen protein Alta1 and previous studies performed in A. alternata (and homologs in other fungal species) instead of paragraphs about WD40 and circadian regulation of host immunity – discussion of the host interactors can be in the discussion. You could move some of the discussion text on Alta1 to the intro. Much work has been done to identify and characterize the role of Alta1 in respiratory inflammatory diseases but this is one of the first comprehensive studies to characterize its role in plant pathogenesis – this should be emphasized. Garrido-Arandia et al. 2016 (https://www.nature.com/articles/srep33468) did show its capacity to bind flavonoids and proposed a plant virulence mechanism based on its structure, and Gomez-Casado et al. 2014 (https://www.sciencedirect.com/science/article/pii/S0014579314001641) demonstrated its ability to bind to and inhibit PR5! Much work done on homologs in plant pathogens e.g. characterization of the Verticillium Alta1 homolog PevD1.

A: Thank you for the suggestion. We have removed the paragraphs about WD40 and circadian regulation of host immunity. And the discovery of the allergen protein Alta1 and previous studies of *A. alternata* and homologs in other fungal species was added in the new revision (Lines 95-106). We modified the introduction as follows:

The allergen protein Alta1 (*Alternaria alternata* allergen 1; Pfam: PF16541), derived from *Alternaria* species, is widely distributed and conserved among the Dothideomycetes and Sordariomycetes [18] and is involved in spore germination [19]. Much work has been done to identify and characterize the role of Alta1 in respiratory inflammatory diseases [20]. Notably, in recent years, a few studies shown that Alta1 plays a significant role in plant pathology. A previous study has demonstrated the capacity of Alta1 to bind to flavonoids and proposed a plant virulence mechanism based on its structure [21]. Likewise, studies have demonstrated its ability to bind to and inhibit pathogenesis-related-5 protein [22]. In addition, a few studies have been performed on homologs in other plant pathogens, including the characterization of the *Verticillium* Alta1 homolog PevD1 [23-27]. However, the role of Alta1 in plant pathogenesis remains largely unknown.

[20] Hernandez-Ramirez G, Pazos-Castro D, Gonzalez-Klein Z, Resuela-Gonzalez JL, Fernandez-Bravo S, Palacio-Garcia L, et al. Alt a 1 promotes allergic asthma in vivo through TLR4-Alveolar macrophages. Front Immunol. 2022;13:877383. Epub 2022/07/19. doi: 10.3389/fimmu.2022.877383. PubMed PMID: 35844541; PubMed Central PMCID: PMCPMC9280186.

[21] Garrido-Arandia M, Silva-Navas J, Ramirez-Castillejo C, Cubells-Baeza N, Gomez-Casado C, Barber D, et al. Characterisation of a flavonoid ligand of the fungal protein Alt a 1. Sci Rep. 2016;6:33468. Epub 2016/09/17. doi: 10.1038/srep33468. PubMed PMID: 27633190; PubMed Central PMCID: PMCPMC5025882.

[22] Gomez-Casado C, Murua-Garcia A, Garrido-Arandia M, Gonzalez-Melendi P, Sanchez-Monge R, Barber D, et al. Alt a 1 from Alternaria interacts with PR5 thaumatin-like proteins. FEBS Lett. 2014;588(9):1501-8. Epub 2014/03/20. doi: 10.1016/j.febslet.2014.02.044. PubMed PMID: 24642375.

-Could introduce the concept of “effector-triggered susceptibility”

A: Thank you for the suggestion. We introduced the concept of “effector-triggered susceptibility” in lines 70-72. As follows:

Pathogens release effectors against host cells to invade plants and suppress PTI [7,8], thereby hijacking plant cellular processes to promote pathogen growth and spread, and this phenomenon known as effector-triggered susceptibility.

-I would suggest removing any mention of the host targets and moving it to the discussion.

A: Thank you for your suggestion, we have removed any mention of the host targets and moved it to the discussion. Additionally, we have added some supplements and modifications (lines 595-603, 618-623 in the new revision).

Lines 595-603: WD40 proteins contain WD repeat motifs, which are protein sequences composed of 44-60 amino acid residues, and serve as a scaffold for the interaction between two proteins or between proteins and DNA [44]. In addition, these motifs play important roles in many fundamental biological processes such as signal transduction, histone modification, DNA damage response, transcription regulation, RNA processing, protein degradation, and apoptosis [45]. Consistent with their essential roles, several WD40 proteins trigger immune responses in humans and mammals [46,47]. In plants, WD40 protein family play important role in the transcriptional regulation of many processes, including plant growth and stress resistance after being inducted by hormones and the circadian clock [48-53].

[44] Xu C, Min J. Structure and function of WD40 domain proteins. Protein Cell. 2011;2(3):202-14. Epub 2011/04/07. doi: 10.1007/s13238-011-1018-1. PubMed PMID: 21468892; PubMed Central PMCID: PMCPMC4875305.

[45] Yan C, Hang J, Wan R, Huang M, Wong C, Shi Y. Structure of a yeast spliceosome at 3.6-angstrom resolution. Science. 2015;349(6253):1182–1191. Epub 2015/08/20. doi: 10.1126/science.aac7629. PubMed PMID: 26292707

[46] Tanti GK, Goswami SK. SG2NA recruits DJ-1 and Akt into the mitochondria and membrane to protect cells from oxidative damage. Free Radic Biol Med. 2014;75:1-13. Epub 2014/07/19. doi: 10.1016/j.freeradbiomed.2014.07.009. PubMed PMID: 25035075.

[47] Tanti GK, Pandey S, Goswami SK. SG2NA enhances cancer cell survival by stabilizing DJ-1 and thus activating Akt. Biochemical and Biophysical Research Communications. 2015;463(4):524-31. doi: 10.1016/j.bbrc.2015.05.069.

Lines 618-623: The temporal control of defense mediated by the circadian clock is represented by rhythmic changes in the levels of defense-related molecules, reflecting the role of the circadian clock in anticipating likely attacks from pathogens [56-58]. For example, in the absence of pathogens, the levels of some defense-related genes, including *FLS2*, *MKK4*/*MKK5*, *MPK3*/*MPK6* and *WRKY22*, and the production of defense signaling molecules, such as SA, JA, and ROS, oscillate with changing peaks throughout the day [59-61].

Lines 52-3 “pathogen-associated molecular patterns”

A: Thank you for your suggestion. We have fixed it (line 65).

Line 59 – re-phrase this- maybe remove “at this stage” and change to plants “have evolved” resistance (R) genes.

A: Thank you for your comment. The sentence has been changed to “Plants have evolved resistance (R) genes…” (line 73 in the new revision).

Line 68 – include a reference for “leading to severe yield losses in chrysanthemum production every year”

A: Thank you for the suggestion. We have added the reference as follows (line 81).

[11] Liu Y, Xin J, Liu L, Song A, Guan Z, Fang W, et al. A temporal gene expression map of Chrysanthemum leaves infected with *Alternaria alternata* reveals different stages of defense mechanisms. Hortic Res. 2020;7:23. Epub 2020/03/07. doi: 10.1038/s41438-020-0245-0. PubMed PMID: 32140232; PubMed Central PMCID: PMCPMC7049303.

Line 109- you cannot claim that Alta1 is indispensable for virulence, since disease symptoms still occurred:

“The Δalta1 mutant strain produced mild disease symptoms on chrysanthemum leaves compared to the wild-type strain (Fig 2B), with a lesion area approximately reduced by 70% (Fig 2C).”

A: Thank you for your comment. We altered “indispensable” into “crucial” (line 110).

Methods:

- Methods (using data from prev paper) – candidate effector identification from RNAseq data. I was surprised that authors didn’t use EffectorP to predict – probably missed many “unique” effectors for Alternaria that are small secreted proteins, since methodology relied upon comparison of gene and domain sequences within existing databases. Were they looking for conserved/”core” effectors? If so, this should be clarified! This is not the current study, so cannot change methods of course. A screen for effectors inducing programmed cell death was then performed through transient expression in Nicotiana – ultimately they are trying to identify conserved effectors that trigger susceptibility?

A: Thank you for your comment. Since effectors often show significant expression differences in the early stages of pathogen infection of plants, RNA-seq analysis has become an effective high-throughput method for identifying these effectors. A screen for effectors in Nicotiana through transient expression to further verify the potential functions of the candidate effectors. To be honest, we have not only screened for effectors that induce programmed cell death but also for those that inhibit it. Here, we focused on effectors that cause hypersensitive response, trying to identify conserved effectors that regulate *A. alternata* infections. In the future, EffectorP will be used to screen some “unique” effectors.

Results:

- Alta1 appears to be important for normal fungal physiology since the knockout has reduced fitness in the form of slower radial plate growth – role in “normal” fungal physiology could be discussed below.

A: Thank you for your comment. We have added the corresponding discussions as follows (lines 551-553).

Strikingly, Alta1 appears to be required for normal fungal physiology, as the Alta1 KO strain exhibited reduced fitness in the form of slower radial plate growth than that of the WT strain (Fig 2A).

- A key result is that the absence of Alta1 leads to reduced disease symptoms and this is not discussed further – is the triggering of programmed cell death promoting the proliferation of Alternaria? Or is the fitness penalty caused by loss of Alta1 leading to reduced disease?

A: Thank you for your comment. We have added the corresponding discussions as follows (lines 553-563).

Additionally, the *Δalta1* mutant exhibited markedly reduced virulence on chrysanthemum compared with that displayed by the WT and complemented strain *Δalta1*/Alta1 (Figs 2B and 2C), suggesting that *Aa*Alta1 has complicated pathogenic roles in the infection process. The fitness penalty caused by loss of Alta1 may be related to the reduced disease severity, indicating Alta1 may be involved in the triggering programmed cell death, which, in turn, promoted the proliferation of the necrotrophic fungus *A. alternata*. In general, the contribution of necrosis-inducing proteins to the virulence of necrotrophic fungi is attributed to their ability to supply nutrients for fungal growth [35]. *Aa*Alta1 induced cell death in a dose-dependent manner (Fig 1B), so indicating that it mainly play a toxic role and promotes *A. alternata* infection by accumulating during the late stages of disease.

- Wonderful work elucidating the mechanism of triggering PCD – I am not an expert in these assays but I know they are extremely time-consuming, and they have been all been performed with appropriate controls, replicates and statistical methods of assessments.

- Is there a homolog of this WD40 protein in humans/mammals too (triggering immune response)?

A: Thank you for the suggestion. In humans and mammals, there are also homologs of WD40 proteins that are related to triggering immune responses. Studies have shown that SG2NA can protects cells from oxidative stress and function in cancer cell proliferation [46,47] (lines 600-601).

[46] Tanti GK, Goswami SK. SG2NA recruits DJ-1 and Akt into the mitochondria and membrane to protect cells from oxidative damage. Free Radic Biol Med. 2014;75:1-13. Epub 2014/07/19. doi: 10.1016/j.freeradbiomed.2014.07.009. PubMed PMID: 25035075.

[47] Tanti GK, Pandey S, Goswami SK. SG2NA enhances cancer cell survival by stabilizing DJ-1 and thus activating Akt. Biochemical and Biophysical Research Communications. 2015;463(4):524-31. doi: 10.1016/j.bbrc.2015.05.069.

Discussion:

- Alta1 is an example of necrotrophic effector-triggered susceptibility?

A: Alta1 ribonuclease secretion contributes to *A. alternata* virulence and induces plant cell death. Therefore, it is an example of necrotrophic effector-triggered susceptibility.

- How should this knowledge be used to breed for disease resistance/tolerance in chrysanthemum?

A: Thank you for your comment. We can introduce *Cm*WD40 into chrysanthemum through genetic engineering method to enhance their disease resistance.

Line 471-2 – “which also suggested that different expressions indicated of AaAlta1 may influence the virulence of A. alternata” – perhaps remove or re-word this.

A: Thank you for your suggestion. We have removed the sentence.

Line 473-4 – “suggesting that AaAlta1 has complicated pathogenic roles in infection” – perhaps expand on this…

A: Thank you for your suggestion. The sentence has been changed as follows (lines 553-556).

The fitness penalty caused by the loss of Alta1 may be related to the reduced disease severity, indicating Alta1 may be involved in triggering programmed cell death, which, in turn, promotes the proliferation of the necrotrophic fungus *A. alternata*.

Line 501 – “serval” – wrong word?

A: We have corrected “serval” into “several”.

Data availability:

 Provide raw data from all experiments in a public archive like figshare.com E.g. raw gel images, yeast two-hybrid data, bimolecular fluorescence complementation data, pull-down assay data, qPCR expression data etc.

A: Thank you for the suggestion. We have uploaded the raw data in figshare (<https://doi.org/10.6084/m9.figshare.27929355.v2> ).

 Sequencing data - BioProject IDs cannot be found – not publicly available yet? Make sure that they are made available prior to publishing.

A: Thank you for the suggestion. We have uploaded the sequencing data and publicly available (NCBI repository, bioproject PRJNA1110193 and PRJNA1110220.).

Reviewer #3: Line 133: “Purified Alta1 protein was tested for cell death activity by infiltrating 300 nM to 3 μM protein solution into the mesophyll of N. benthamiana leaves”. How can the authors be sure that the protein solution was infiltrated exactly into the mesophyll layer?

A: Thank you for your comment. By using a needle-free syringe to create a pressure difference, the protein solution is forced into the mesophyll layer. It was determined whether the protein solution has successfully entered the mesophyll layer based on the visible water-soaked spot in the infiltrated area.

Line 144: “The ability of expression Alta1ΔSP to induce cell death was significantly reduced in N. benthamiana. In contrast, expression of Alta1 did trigger cell death (Fig 1C), suggesting that the ability of AaAlta1 to induce cell death are relyies on the SP”

I don’t fully agree with that statement. The SP is not required to cell death induction but only to protein secretion. However, when looking at Fig. 1C, necrotic tissue is visible at the infiltration spot. Can you please elaborate on that?

A: Thank you for your comment. We have modified this statement (lines 184-185 in the new revision). The ability of expression *Aa*Alta1 to induce cell death is not entirely dependent on SP, Alta1 may possess specific regions that might help to enter into plant cells. This still requires further research in the future.

In Fig. 1A it is not clear whether the mature effector does consist only in the Allergene motif highlighted in green or there are other motifs which required (or not) for effector activity in terms of interaction with CmWD40 and cell death induction?

A: Thank you for your comment. We verified the interactions between *Cm*WD40 and different fragments of *Aa*Alta1, as well as identified Alta1 fragments that induced cell death (S9 Fig). The Y2H assay verified the interactions between CmWD40 and the deletion variants of the *Aa*Alta1 protein containing the *A. alternata* allergen motif, including *Aa*AltA1^SP+42-145^, *Aa*AltA1^SP+42-145^ and *Aa*AltA1^SP+42-145^ (S9A Fig). To define the region required for the cell death-inducing activity of Alta1, we generated a series of N-terminal and C-terminal deletion variants of Alta1 and tested their cell death-inducing activity (S9B Fig). When the allergen motif was present, the deletion variant AaAltA1^SP+42-145^ retained full activity. However, when residues 43-145 were deleted, the cell death-inducing activity was lost. Taken together, these data illustrated that mature effector is only present in the green-highlighted allergen motif, which is indispensable for the interaction with *Cm*WD40 and induction of cell death. Please see in lines 308-320.

S9 Fig. (A) Yeast two‐hybrid assay demonstrates protein-protein interactions between *Cm*WD40 and different fragments of *Aa*Alta1. SD-4, SD/−His/−Leu/−Trp/−Ade- medium; SD-2, SD/−Leu/−Trp- medium. (B) Images showing the phenotypes of leaves infiltrated with Agrobacterium strains harboring constructs encoding the different deletion fragments of AaAlta1 3 days after infiltration.

In Fig. 1 description, line 185. I guess the authors meant that the SP of PR1 protein served as positive control.

A: Thank you for your suggestion. We have provided a more accurate explanation: PR1 is the signal peptide from pathogenesis-related protein 1 protein and used as a positive control (lines 158-159).

Line 207 mentions that “These results demonstrate that Alta1 is an essential virulence factor during the A. alternata infection stage”. I wonder which stage do you mean here.

A: Thank you for your comment. It’s the early stage of *A. alternata* infection. The sentence has been changed to “These results demonstrate that Alta1 is an essential, functionally conserved virulence factor required during the early stage of *A. alternata* infection” (lines 234-236).

Line 227: from your transcriptome analysis it is not clear whether the authors did check DEGs just from infiltrated tissue or from Alta1 pre-infiltrated tissue subsequently inoculated with the fungus.

Moreover, would it be possible to have some information on DEGs host genes in inoculated vs non-inoculated tissue?

A: Thank you for your comment. Transcriptome analysis did check DEGs just from infiltrated tissue. We made the following modifications:

To investigate the molecular mechanisms underlying Alta1-induced resistance in chrysanthemums plants, RNA‐seq analysis was performed on tissues infiltrated with purified AaAlta1 and buffer (mock). The outcome was a set of 26,884 DEGs in Alta1-treated chrysanthemum leaves, of which 13,769 DEGs were upregulated, and 13,115 DEGs were downregulated (Fig 3C) (lines 278-282).

Moreover, we analyzed the host genes of DEGs in both inoculated and non-inoculated tissues of WT chrysanthemum, and compared the transcriptomes of OX-CmWD40 and WT chrysanthemum leaves with or without *A. alternata* inoculation. The Venn diagram shows that Alta1 protein and *A. alternata* co-induce 250 upregulated DEGs (S17A Fig). The KEGG enrichment analysis showed that up-regulated DEGs were significantly enriched in ‘MAPK signaling pathway-plant’, ‘Plant-pathogen interaction’, ‘Plant hormone signal transduction’, ‘alpha-linolenic acid metabolism’ and ‘isoflavonoid biosynthesis’ (S17B Fig). This result suggested that inoculation induced an immune response similar to that of the Alta1-treatment and that these 250 genes are pivotal to elucidating Alta1-induced defense responses against *A. alternata*. Please see in lines 570-573.

S17 Fig. Differentially expressed genes (DEGs) co-upregulated by Alta1 and *Alternaria alternata* infection. (A) Venn diagram analysis of the DEGs induced by Alta1 and *A. alternata*. (B) Kyoto Encyclopedia of Genes and Genomes enrichment analysis of the DEGs co-upregulated by Alta1 and *A. alternata*.

In Fig. 3A, I don’t entirely understand which is the difference between Mock and Control. Moreover Fig. 3D shows increased transcript levels of isoflavonoid biosynthesis related genes. These might represent important phytoalexins. Do the authors think that it might be worthy to elaborate on that?

A: Thank you for your comment. Control was merely a reference for a normal leaf, and to avoid ambiguity, we have removed it from Fig. 3A. Mock is a blank control for Alta1.

Additionally, we have added KEGG enrichment for isoflavonoid biosynthesis in the results (see in lines 284-285). And an elaborate discussion regarding isoflavonoid biosynthesis and phytoalexins has been added in lines 573-587, to extend the implication of our findings in different signaling pathways.

“In addition, the Alta1treatment upregulated the transcript levels of genes implicated in isoflavonoid biosynthesis (Fig 3D), including those encoding the cytochrome P450, pterocarpan synthase, and 2-hydroxyisoflavanone dehydratase enzyme, among others (S18 Fig). Isoflavones are a group of phenolic compounds, which mediate important interactions with plant-associated microbes, including in defense against pathogens. In addition, in certain cases, isoflavonoids can act as phytoalexins, providing resistance against infections. For instance, a new isoflavonoid, triticein, catalyzed by a cytochrome P450 enzyme, inhibits fungal spore germination and bacterial growth [40]. In soybeans, glyceollin is a key phytoalexin induced by cell wall glucan elicitors, used to resist infection from *P. sojae* [41]. In *Medicago truncatula*, medicarpin works synergistically with SA to combat *Erysiphe pisi* and *Rhizoctonia solani* [42]. Based on these results, we inferred that Alta1-induced genes encode enzymes that catalyze various steps in the biosynthetic pathway leading to the formation of isoflavonoids which are involved in the production of phytoalexins in certain plants, which, in turn, play a role in plant defense against pathogens.”

S18 Fig. Heatmaps of differentially expressed genes (DEGs) encoding isoflavonoid biosynthesis-related genes in RNA-seq. The gene expression value was normalized Log2(FPKM [fragments per kilobase of transcript per million fragments mapped] + 1). From left to right are mock and Alta1. Mock, heat map of the DEGs in mock-infiltrated tissues; Alta1, heat map of the DEGs in 300 nM Alta1-infiltrated tissues.

[40] Polturak G, Misra RC, El-Demerdash A, Owen C, Steed A, McDonald HP, et al. Discovery of isoflavone phytoalexins in wheat reveals an alternative route to isoflavonoid biosynthesis. Nat Commun. 2023;14(1):6977. Epub 2023/11/02. doi: 10.1038/s41467-023-42464-3. PubMed PMID: 37914713; PubMed Central PMCID: PMCPMC10620232

[41] Ng TB, Ye XJ, Wong JH, Fang EF, Chan YS, Pan W, et al. Glyceollin, a soybean phytoalexin with medicinal properties. Appl Microbiol Biotechnol. 2011;90(1):59-68. Epub 2011/02/22. doi: 10.1007/s00253-011-3169-7. PubMed PMID: 21336922.

[42] Gupta A, Awasthi P, Sharma N, Parveen S, Vats RP, Singh N, et al. Medicarpin confers powdery mildew resistance in Medicago truncatula and activates the salicylic acid signalling pathway. Mol Plant Pathol. 2022;23(7):966-83. Epub 2022/03/10. doi: 10.1111/mpp.13202. PubMed PMID: 35263504; PubMed Central PMCID: PMCPMC9190973.

Line 276: the authors previously mentioned that the SP is required for effector activity. I wonder how the effector can now localize at the nucleus and interact with CmWD40 if expressed without SP. Could the author elaborate on that? how was the localization experiment conducted?

A: Thank you for your comment. We have revised this statement based on our data. The SP is not required for effector activity but only to protein secretion.

Regarding the subcellular localization experiment of Alta1, we generated another construct encoding *Aa*Alta1 without the SP (*Aa*Alta1ΔSP) fused to GFP, *Aa*Alta1ΔSP-GFP. We investigated the subcellular localization of *Aa*Alta1ΔSP-GFP in *N. benthamiana* cells by confocal microscopy.

We independently validated their interaction in plant cells by dual-vector bimolecular fluorescence complementation (BiFC) assay using the pSPYNE(173) and pSPYCE(M) vectors. In this assay, we fused *Cm*WD40 to the pSPYNE(173) vector (pSPYNE -*Cm*WD40) and AaAlta1 to the pSPYCE(M) vector (pSPYCE-*Aa*Alta1) (Fig 4C). We then transiently expressed these constructs by *Agrobacterium*-mediated co- infiltration with the nuclear marker D53-RFP into *N. benthamiana* leaves. We observed the reconstitution of Venus fluorescence in the nucleus of leaf epidermal cells, indicating that *Aa*Alta1 and *Cm*WD40 interact in the plant cell nucleus.

Fig. 4. Overall, the figure panel do not match with the legend. Can this please be adjusted?

A: Thank you for the suggestion. We have adjusted the order of the legends.

In Fig. 5, I wonder if it would be wise to combine the graph in Fig S6 with the two graphs shown in Fig. 5A and B. Would it make sense to plot both transcripts level of CmWD40 in mock plants and in infected plants either as line or as histogram (Fig 5B)?

A: Thank you for the suggestion. We have combined the graph from Fig S6 with the one shown in Fig. 5A, and made corresponding modifications to the legend of Fig. 5A (Lines 365-373).

Additionally, we plotted the transcript levels of *Cm*WD40 in both mock and infected plants as lines. qRT-PCR analysis was carried out to determine the mRNA levels of *Cm*WD40 at various stages of infection in hosts, preliminary investigated whether *Cm*WD40 has an important role in the interaction between *A. alternata* and the host.

Fig 5. *Cm*WD40 regulates the resistance of chrysanthemum plants to *Alternaria alternata*. (A) The rhythmic expression pattern of CmWD40 and the temporal variation in chrysanthemum susceptibility to *A. alternata*. The line chart represents the rhythmic expression pattern of *Cm*WD40 in wild-type (WT) plants under long-day (LD) conditions. Leaves were collected every 6 h at the indicated times, and gene expression levels were assessed using quantitative reverse transcriptase polymerase chain reaction (qRT-PCR). The bar chart represents the mean lesion size on chrysanthemum leaves inoculated with *A. alternata* at dawn, i.e., at zeitgeber time (ZT)0 and every 6 h for 48 h under LD conditions. The size of the lesions was statistically analyzed at 48 hpi. Data are presented as the mean ± standard error of three biological replicates. (B) Relative expression levels of the *Cm*WD40 gene during infection were assessed using qRT-PCR. Data are presented as the mean ± standard error of three biological replicates. Different letters at the top of error bars indicate significant differences (*P* < 0.05, Tukey’s test).

Fig. 5E D and H should indicate the time point at which transcripts level were measured.

A: Thank you for the suggestion. We have added the time point at which transcript levels were measured in the legends of Figs 5E and 5H (see in lines 381-382, 389). We have also added the time points for measuring protein levels in the legend of Fig D (see in line 378-379).

Line 484: Since Alta1 also caused necrosis in *N. benthamiana*, it would be wise to test the interaction between Alta1 and NbWD40?

A: Thank you for the suggestion. We performed a Y2H assay using *Nb*WD40 fused to the activation domain (AD) of the yeast transcriptional activator Gal4 and *Aa*Alta1 fused to its DNA-BD. The results showed *Nb*WD40 interaction with *Aa*Alta1 in yeast (S10 Fig). Please see in lines 317-318.

S10 Fig. Yeast-two-hybrid assays showing that *Aa*Alta1 interacts with *Nb*WD40. SD-4, SD/−His/−Leu/−Trp/−Ade- medium; SD-2, SD/−Leu/−Trp- medium.

In Material and Methods, I would suggest including information about the A. alternata isolate used. Isolate name/code, where it was collected and so on.

A: Thank you for the suggestion. We added relevant information. Please see in lines 684-687: The pathogen test strain *A. alternata* was isolated and identified from typical diseased leaves of ‘Fubaiju’, a cultivar found in the chrysanthemum tea-producing area of Futianhe Town, Macheng City, Hubei Province, China in 2017.

Line 594: How do you mean “plants were inoculated with a quantitative amount of mycelium”?

A: Thank you for your comment. The strain *A. alternata* was cultured in 200 mL of potato dextrose broth liquid medium on a 180 r/min shaker for 24 h. Then, 1 mL homogenous mycelium suspension was collected (the amount of mycelium contained in each milliliter of suspension was constant), used a fine-bristle brush to pick out the mycelium to inoculate. (lines 693-695 in the new revision)

Line 701: the “previously described chrysanthemum transformation method” requires a reference.

A: Thank you for the suggestion. We added a reference. See in line 858.

[79] Wang J, Guan Y, Ding L, Li P, Zhao W, Jiang J, et al. The *CmTCP20* gene regulates petal elongation growth in *Chrysanthemum morifolium*. Plant Sci. 2019;280:248-57. Epub 2019/03/03. doi: 10.1016/j.plantsci.2018.12.008. PubMed PMID: 30824003.

As a final curiosity I’d like to ask the following question:

How conserved is Alta1 effector within the genus Alternaria? Can you speculate on its role in other pathosystems?

A: We have done the comparison of homologous proteins in other *A. alternata* strains using by MSA (S3 Fig). We found the homology of these proteins is higher than 86%, suggesting that this gene and its encoded protein is high conserved (lines 131-136 in the new revision).

Additionally, much work has been done on Alta1 homologs in plant pathogens, such as the characterization of the Verticillium Alta1 homolog PevD1 and *Magnaporthe oryzae* Alta1 homolog *Mo*Hrip1. From these studies, we think that the function of Alta1 is conserved. Therefore, we speculate that Alta1 effector has similar functions in other pathosystems. It contributes to phytopathogen virulence and induces plant immune responses.

S3 Fig. Multiple sequence alignment of *Aa*Alta1 with 11 orthologs from other *Alternaria alternata* strains. *Alternaria arborescens* (XP_028503382.1), *Alternaria gaisen* (KAB2107613.1), *Alternaria burnsii* (XP_038782547.1), *Alternaria panax* (KAG9189691.1), Alternaria atra (XP_043172693.1), *Alternaria dauci* (XP_069306948.1), *Alternaria solani* (WPZ50800.1), *Alternaria hordeiaustralica* (XP_049246647.10), *Alternaria metachromatica* (XP_049193700.1), *Alternaria incomplexa* (XP_051290017.1), *Alternaria irosae* (XP_046029020.1).
