# Supplementary material for: Alternaria alternata effector AaAlta1 targets CmWD40 and participates in regulating disease resistance in Chrysanthemum morifolium
Source: PLoS Pathog. 2025 Mar 31;21(3):e1012942. doi: 10.1371/journal.ppat.1012942 (PMC11957361; doi:10.1371/journal.ppat.1012942)
Supplement: Attachment — Submitted filename: Response_to_reviewers_auresp_2.docx [file ppat.1012942.s023.docx]

Dear. Dr. Thomma,

Thank you very much for sending our manuscript for peer review. At the same time, we are very grateful to the reviewer for carefully reviewing our manuscript and putting forward the valuable comments and suggestions.

After carefully reading the reviewer's comments, we find that the comments are of great help for the improvement of our manuscript. In the past days, we have made great efforts to carefully revise the manuscript according to these comments. All of the comments have been considered in the revised manuscript, and all of the revisions are marked in red in the revised version.

Below we have provided in detail the point-by-point replies to the reviewer’s comments. Now we are resubmitting the revised manuscript to ***PLOS Pathogens***, and sincerely hope that it can be finally recommended for publication in this journal after the further evaluation.

Sincerely,

Corresponding author:

Jiafu Jiang

Email: jiangjiafu@njau.edu.cn

-----------------------------------------------------------------------------------------------------

REVIEWER COMMENTS:

**Part I - Summary**

**Please use this section to discuss strengths/weaknesses of study, novelty/significance, general execution and scholarship.**

Reviewer #3: Dear Authors,

after reviewing the second version of the manuscript, I appreciate the effort you put to address my concerns by conducting additional experiments to strengthen the validity of your results. Most of them have been clearly elucidated and added very interesting aspects to the research you conducted.

However, I have to point out that the section Discussion appears to me as a summary of the findings listed in the section results. I don't see any attempt to elaborate on the biological meaning of your scientific discoveries and to connect the information you collected. The manuscript shows a considerable amount of interesting data but, in my opinion, there is no biological story line that can shed light on the mode of action of the effector protein in a contest of plant immunity. Also, I don't see any proposed follow-up research approach to investigate the biological function of the Alta1 effector in the manipulation of JA-dependent host defense responses.

For example, as follow-up research question it would be interesting to investigate how the JA related defense responses are affected by the interaction between Alta1 and the clock regulatory element CmWD40.

A: Thank you for your suggestion. Following your suggestions, we have supplemented and revised the discussion section. Please see in lines 530-686.

**Part II – Major Issues**

Reviewer #3: If the A. alternata Alta1 K.O. lines exhibit reduced radial growth on plates, do you find reasonable to assume that the reduction in lesion size observed on inoculated plants is largely related to the contribution to fungal virulence of the investigated effector?

A: Thank you for your comment. Because the lesion area of both *Δalta1* mutant strains was reduced by about 70% compared with that of the WT strain (Fig 2C), we supplemented the experiment of inoculating chrysanthemum leaves with wild-type strains at normal mycelial amounts and *alta1* mutant strains at double mycelial amounts. The results showed that the pathogenicity of the mutant strains with double inoculum was still lower than that of the wild-type strains, with a reduction of about 65% in lesion area, further demonstrating that the reduction in lesion size observed on inoculated plants was largely related to the contribution of Alta1 to fungal virulence (S6 Fig). Please see in lines 226-230.

S6 Fig. The reduced virulence of *Δalta1* mutants is not largely due to a decrease in growth.

Maybe I misunderstood that but you often stated that your findings indicate that the AaAlta1 – CmWD40 – CmMYC2 interaction positively regulates chrysanthemum resistance to A. alternata. But then why is the fungus less virulent when you knock out Alta1? What does this contribution rely on?

A: Alta1 exhibits dual functions in plant–pathogen interactions. On the one hand, Alta1 was involved in the pathogenic signaling pathways of *A. alternata*，and on the other hand, it activates innate immune response in plants. This seemingly paradoxical phenomenon is actually not untraceable. In the past few decades, as research on plant pathogens has progressed, several effectors have been thoroughly studied. As key virulence determinants, these effectors are capable of causing host plant tissue death and facilitating host infection, all the while triggering plant immune responses. For example, a GH12 protein, XEG1, secreted by *P. sojae* induces cell death, triggers plant innate immune responses, and degrades the plant cell wall through its enzymatic activity to promotes pathogen colonization (Ma et al. 2015); *C. shiraiana* secretes the apoplastic effector Cs02526, which induced strong immune responses in plants but also improved pathogenicity, facilitating *C. shiraiana* infection (Zhang et al., 2024); as effectors, necrosis and ethylene-inducing peptide 1-like proteins (NLPs) act as toxin-like virulence factors that induce tissue necrosis and trigger plant immune responses (Lian et al. 2022; Pirc et al. 2023). These findings potentially explain the different function of Alta1 in pathogenic mechanisms and the induction of immune responses through distinct signaling pathways. It acts as an important virulence factor during *A. alternata* infection but also acts as an effector in chrysanthemum, where it can trigger defense responses. In this study, what we have elucidated is the signaling pathway by which Alta1 induces plant disease resistance responses, while the molecular mechanisms underlying its contribution to virulence remain unclear.

Ma Z, Song T, Zhu L, Ye W, Wang Y, Shao Y, et al. A Phytophthora sojae Glycoside Hydrolase 12 Protein Is a Major Virulence Factor during Soybean Infection and Is Recognized as a PAMP. Plant Cell. 2015;27(7):2057-72. Epub 2015/07/15. doi: 10.1105/tpc.15.00390. PubMed PMID: 26163574; PubMed Central PMCID: PMCPMC4531360.

Zhang S, Li R, Fan W, Chen X, Liu S, Zhu P, et al. Effector Cs02526 from Ciboria shiraiana induces cell death and modulates plant immunity. Plant Physiol. 2024;196(1):579-91. Epub 2024/05/16. doi: 10.1093/plphys/kiae286. PubMed PMID: 38753366; PubMed Central PMCID: PMCPMC11376374.

Lian J, Han H, Chen X, Chen Q, Zhao J, Li C. Stemphylium lycopersici Nep1-like Protein (NLP) Is a Key Virulence Factor in Tomato Gray Leaf Spot Disease. J Fungi (Basel). 2022;8(5). Epub 2022/05/29. doi: 10.3390/jof8050518. PubMed PMID: 35628773; PubMed Central PMCID: PMCPMC9144795.

Pirc K, Albert I, Nurnberger T, Anderluh G. Disruption of plant plasma membrane by Nep1-like proteins in pathogen-plant interactions. New Phytol. 2023;237(3):746-50. Epub 2022/10/11. doi: 10.1111/nph.18524. PubMed PMID: 36210522; PubMed Central PMCID: PMCPMC10100409.

Yang B, Wang Y, Tian M, Dai K, Zheng W, Liu Z, et al. Fg12 ribonuclease secretion contributes to Fusarium graminearum virulence and induces plant cell death. J Integr Plant Biol. 2021;63(2):365-77. Epub 2020/07/30. doi: 10.1111/jipb.12997. PubMed PMID: 32725938.

Based on your comments in the rebuttal letter, unfortunately I still not entirely understand how the SP of the effector protein could be involved in triggering host cell death.

Line 187 "In contrast, the expression of Alta1 did trigger cell death (Fig 1C), suggesting that Alta1 may possess specific regions that may help to enter into plant cells." this statement does not seem to be related to cell death inducing capability of Alta1. Moreover, how does it trigger cell death if the plant exhibit enhanced resistance upon Alta1 overexpression? Is a certain JA gradient involved in triggering host cell death?

A: Thank you for pointing this out. The SP is a key structure in effector proteins that guides their secretion. Effectors are typically secreted into the extracellular space via the signal peptideto exert their functions. Therefore, the SP plays an important role in enabling effectors to exert their functions further. This revised statement as follows (Lines 187-188):

The ability of expression Alta1^ΔSP^ to induce cell death was significantly reduced in *N. benthamiana* (Fig 1C), suggesting that Alta1 may be targeted to the extracellular space or cells of *N. benthamiana* tissue to induce cell death.

Effector proteins may be recognized by plant resistance proteins either directly or indirectly, triggering a typical effector-triggered immunity (ETI). This leads to a number of overlapping downstream outputs, such as the hypersensitive response (HR), mitogen-activated protein kinase (MAPK) cascades, calcium flux, reactive oxygen species (ROS) burst, transcriptional reprogramming, and phytohormone signaling (Thulasi et al., 2018; Tsuda et al., 2010; Peng et al., 2017). For instance, a previous study reported that the *B. graminis* effector AvrPm2 was recognized by the wheat resistance protein Pm2 (Praz et al. 2017). Alternatively, effector-mediated plant cell death may result from the prolonged effects on its virulence targets (Cunnac et al. 2009). For example, the *P. sojae* extracellular effector XEG1 was identified as a novel PAMP that strongly induces cell death in both host and non-host cells (Ma et al. 2015); a receptor-like protein, PsXEG1, in *N. benthamiana*, was later identified as one that specifically recognizes XEG1 to activate plant immunity (Wang et al. 2018; Sun et al., 2022). Necrosis and ET-inducing peptide 1-like proteins (NLPs) from fungi, bacteria, and oomycetes, have also been reported to exert cytotoxic effects. Binding to glycosyl inositol phosphoryl ceramide sphingolipids, which are abundant in plant plasma membranes, is important for the cytolytic effects of NLPs in eudicots (Lenarčič et al. 2017). Apart from their cytolytic ability, many NLPs also carry a 20 amino acid motif (nlp20) that elicits PTI responses in Arabidopsis and related plant species similar to bacterial flagellin. Based on these researches, we inferred that Alta1 has similar functions. On the one hand, Alta1 induces cell death through some unknown pathogenic pathways, such as promoting the secretion of toxins and cell wall-degrading enzymes by *A. alternata*. On the other hand, chrysanthemum exhibits enhanced resistance upon Alta1 overexpression, suggest that Alta1 can be recognized by chrysanthemum and activate plant immunity.

Additionally, many studies have shown that a certain JA gradient involved in triggering plant immunity response (Zhang et al.,2023). However, there are currently no studies demonstrating that JA directly induces cell death. Our experiments have showed that the increasing ability of the Alta1 protein to induce cell death with increasing concentrations of protein solution from 300 nM to 3 μM (Fig 1B), suggesting that the higher the concentration of Alta1, the stronger the immune response triggered. Further experiments have confirmed that the content of JA gradually increases with the increasing concentration of Alta1 treatment (S8 Fig), suggesting that the immune response induced by Alta1 includes JA signaling. However, it is not clear how Alta1 induces cell death and whether JA is involved in the cell death induced by Alta1. Further research is needed in the future.

Thulasi Devendrakumar K, Li X, Zhang Y. MAP kinase signalling: interplays between plant PAMP- and effector-triggered immunity. Cell Mol Life Sci. 2018;75(16):2981-9. Epub 2018/05/24. doi: 10.1007/s00018-018-2839-3. PubMed PMID: 29789867; PubMed Central PMCID: PMCPMC11105241.

Tsuda K, Katagiri F. Comparing signaling mechanisms engaged in pattern-triggered and effector-triggered immunity. Curr Opin Plant Biol. 2010;13(4):459-65. Epub 2010/05/18. doi: 10.1016/j.pbi.2010.04.006. PubMed PMID: 20471306.

Peng Y, van Wersch R, Zhang Y. Convergent and Divergent Signaling in PAMP-Triggered Immunity and Effector-Triggered Immunity. Mol Plant Microbe Interact. 2018;31(4):403-9. Epub 2017/11/15. doi: 10.1094/MPMI-06-17-0145-CR. PubMed PMID: 29135338.

Praz CR, Bourras S, Zeng F, Sanchez-Martin J, Menardo F, Xue M, et al. AvrPm2 encodes an RNase-like avirulence effector which is conserved in the two different specialized forms of wheat and rye powdery mildew fungus. New Phytol. 2017;213(3):1301-14. Epub 2016/12/10. doi: 10.1111/nph.14372. PubMed PMID: 27935041; PubMed Central PMCID: PMCPMC5347869.

Cunnac S, Lindeberg M, Collmer A. Pseudomonas syringae type III secretion system effectors: repertoires in search of functions. Current Opinion in Microbiology. 2009;12(1):53-60. doi:10.1016/j.mib.2008.12.003.

Ma Z, Song T, Zhu L, Ye W, Wang Y, Shao Y, et al. A Phytophthora sojae Glycoside Hydrolase 12 Protein Is a Major Virulence Factor during Soybean Infection and Is Recognized as a PAMP. Plant Cell. 2015;27(7):2057-72. Epub 2015/07/15. doi: 10.1105/tpc.15.00390. PubMed PMID: 26163574; PubMed Central PMCID: PMCPMC4531360.

Wang Y, Xu Y, Sun Y, Wang H, Qi J, Wan B, et al. Leucine-rich repeat receptor-like gene screen reveals that Nicotiana RXEG1 regulates glycoside hydrolase 12 MAMP detection. Nat Commun. 2018;9(1):594. Epub 2018/02/11. doi: 10.1038/s41467-018-03010-8. PubMed PMID: 29426870; PubMed Central PMCID: PMCPMC5807360.

Sun Y, Wang Y, Zhang X, Chen Z, Xia Y, Wang L, Sun Y, Zhang M, Xiao Y, Han Z, Wang Y, Chai J. Plant receptor-like protein activation by a microbial glycoside hydrolase. Nature. 2022 Oct;610(7931):335-342. doi: 10.1038/s41586-022-05214-x. Epub 2022 Sep 21. PMID: 36131021.

Lenarčič T, Albert I, Böhm H, Hodnik V, Pirc K, Zavec AB, et al. Eudicot plant-specific sphingolipids determine host selectivity of microbial NLP cytolysins. Science. 2017;358(6369):1431-4. doi: doi:10.1126/science.aan6874.

Zhang S, Miao W, Liu Y, Jiang J, Chen S, Chen F, et al. Jasmonate signaling drives defense responses against Alternaria alternata in chrysanthemum. BMC Genomics. 2023;24(1):553. Epub 2023/09/19. doi: 10.1186/s12864-023-09671-0. PMID: 37723458; PubMed Central PMCID: PMCPMC10507968.
